# Dynamic Sub‐Nanoscale “Water Fingers” in Interfacial Polymerization

**DOI:** 10.1002/smll.202504497

**Published:** 2025-06-20

**Authors:** Zhaohuan Mai, Tomohisa Yoshioka, Akshay Deshmukh, Tianmu Yuan, Junyong Zhu, Jinkai Yuan, Ralph Rolly Gonzales, Ayano Yamamoto, Yongxuan Shi, Wenming Fu, Kecheng Guan, Zhan Li, Pengfei Zhang, John H Lienhard, Hideto Matsuyama

**Affiliations:** ^1^ Research Center for Membrane and Film Technology Kobe University Kobe 657‐8501 Japan; ^2^ Graduate School of Science Technology and Innovation Kobe University Kobe 657‐8501 Japan; ^3^ Department of Mechanical Engineering Massachusetts Institute of Technology Cambridge MA 02139‐4307 USA; ^4^ Department of Chemical Engineering The University of Manchester Manchester M1 3AL UK; ^5^ School of Chemical Engineering Zhengzhou University Zhengzhou 450001 P. R. China; ^6^ Laboratoire de Chimie de la Matière Condensée de Paris (LCMCP) Sorbonne Université CNRS UMR 7574 Paris 75005 France; ^7^ Department of Chemical Science and Engineering Kobe University Kobe 657‐8501 Japan

**Keywords:** interfacial polymerization, liquid interface, membranes, molecular dynamics, water structures

## Abstract

Interfacial polymerization (IP) is widely used to fabricate high‐performance membranes, yet the molecular‐level dynamics that govern monomer transport across liquid–liquid interfaces remain poorly understood. Here it is reported that sub‐nanoscale “water fingers”—transient chains of water molecules—modulate the interfacial behavior of amine monomers during IP, dictating the structure and performance of the resulting polyamide films. Using molecular dynamics simulations of archetypal membrane‐forming systems (*m*‐phenylenediamine (MPD)–trimesoyl chloride (TMC) for reverse osmosis and piperazine (PIP)–TMC for nanofiltration), it is revealed that water fingers differentially stabilize monomer transport across the aqueous‐organic interface, correlating with experimentally observed disparities in film density and permeability. These findings offer a new physical picture of interfacial reactivity, establishing water fingers as critical, tunable elements of monomer transport. This work provides mechanistic insights into a century‐old reaction and opens new design strategies for ultrathin films and interfacial materials.

## Graphical abstract

Monomer transport in interfacial polymerization (IP) is governed by free energy barriers and dynamic “water fingers” at the water/*n*‐hexane interface. Molecular simulations reveal that these transient structures selectively drive piperazine (PIP) into the organic phase while confining *m*‐phenylenediamine (MPD) at the interface, dictating nanofiltration (NF) and reverse osmosis (RO) membrane properties. Tuning water fingers enables precise polymer design.

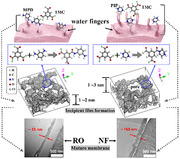

## 1 Introduction

Reactions occurring “on water,” “at the interface,” or via “phase‐transfer” provide versatile platforms for modern chemistry across diverse disciplines,^[^ ^1^, ^2^, ^3^ ^]^ leveraging the unique molecular structures and dynamics of aqueous interfaces.^[^ ^4^, ^5^, ^6^, ^7^, ^8^, ^9^, ^10^ ^]^ These confined environments host phenomena at scales beyond direct visual perception. In 1993, Benjamin introduced a molecular‐level picture of ion transport across water‐immiscible liquid interfaces, explicitly identifying “water fingers” through molecular dynamics (MD) simulations. These transient chains of hydrogen‐bonded water molecules bridge solvation shells from the organic phase into the bulk aqueous phase on picosecond timescales.^[^ ^11^ ^]^ Over subsequent decades, the theoretical existence of such sub‐nanoscale interfacial water fingers has been confirmed,^[^ ^12^, ^13^ ^]^ offering insights into the counterintuitive anomalies of liquid water, which arise from its dynamic hydrogen‐bonding network and interactions with solutes.^[^ ^14^, ^15^ ^]^ Water fingers have since been recognized as key mediators in interfacial solute transport, enabling spontaneous translocation of ions and neutral species across seemingly immiscible interfaces. Consequently, we can no longer be satisfied by a picture that portrays the liquid solvent as a structureless medium, and the impact of aqueous interfaces must be addressed at the microscopic level.^[^ ^1^ ^]^

Despite these advances, the active role of buried aqueous interfaces in interfacial polymerization (IP) remains poorly understood.^[^ ^16^ ^]^ IP, a reaction between two fast‐reacting monomers at the interface of two immiscible phases, forms the polyamide selective layers of thin‐film composite membranes, widely used in reverse osmosis (RO), nanofiltration (NF), and organic solvent separations.^[^ ^17^ ^]^ Classical theories regard IP as a diffusion‐reaction process dominated by simple Fickian diffusion, with no specific orienting or aligning effects attributed to the interface.^[^ ^16^ ^]^ Typically, IP involving *m*‐phenylenediamine (MPD) in water and trimesoyl chloride (TMC) in an organic solvent (usually *n*‐hexane) produces a fully aromatic, highly crosslinked RO membrane. Replacing MPD with piperazine (PIP), an aliphatic diamine, yields a semi‐aromatic NF membrane with markedly different characteristics.^[^ ^18^ ^]^ For instance, NF membranes (pore sizes ≈0.5–2 nm) exhibit an order‐of‐magnitude higher water permeance (up to ≈53.5 L m^−2^ h^−1^ bar^−1^)^[^ ^19^ ^]^ and significantly lower monovalent salt rejection than RO membranes (internal cavity sizes < 0.5 nm).

Although significant experimental efforts have been made to optimize IP membrane performance,^[^ ^20^, ^21^, ^22^, ^23^, ^24^ ^]^ the molecular mechanisms that govern monomer transport and reaction at the water/organic interface are not well characterized—particularly the role of interfacial water structures in guiding monomer migration, primarily due to severe limitations in both spatial and temporal scales imposed by ultrathin film formation.^[^ ^25^ ^]^ Many fundamental questions, including how subtle variations in amine monomer chemistry and structure drive macroscopic disparities in RO and NF performance, remain unanswered.^[^ ^26^ ^]^ Recent work has shown that subtle alterations in solute structure can dramatically alter interfacial orientation and behavior,^[^ ^27^ ^]^ suggesting that aqueous interfaces may play a more active role in polymer formation than previously assumed.^[^ ^27^ ^]^

**Scheme 1:**
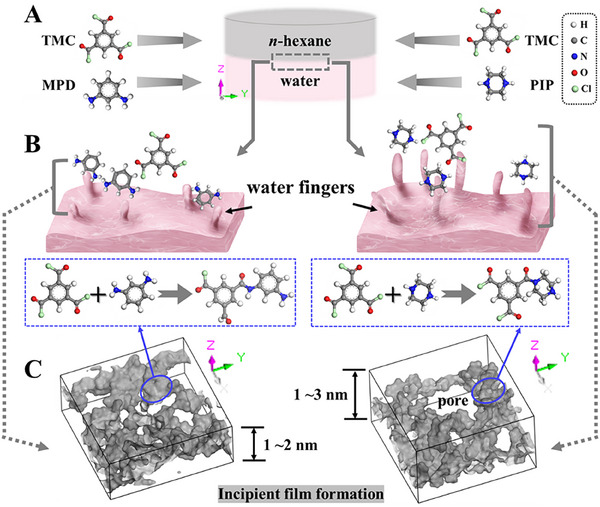
Influence of interfacial water fingers on the incipient film formation of RO (left) and NF (right) membranes. A) IP reactive monomers. B) Water fingers mediate the trans‐interface migration of amine monomers. C) Formation of initial incipient films (dark gray regions).

To address these knowledge gaps, we used MD simulations to model water/*n*‐hexane interfaces and investigate monomer behavior in representative RO and NF membrane systems. Specifically, we analyzed MPD–TMC (aromatic–aromatic) and PIP–TMC (aliphatic–aromatic) systems (Figures S1–S3 and Tables S1–S3, Supporting Information). By decoupling the diffusion and reaction stages, we applied a quasi‐steady‐state approximation^[^ ^28^ ^]^ for interfacial diffusion and an in situ crosslinking protocol to model incipient film formation. We discovered that water fingers—transient, directional hydrogen‐bonded structures—play a critical role in mediating the trans‐interface migration of amine monomers. These structures differ in orientation and spatial distribution depending on monomer chemistry and hydrophilicity. Our findings demonstrate that the biphasic interface actively modulates monomer availability and orientation through structured water pathways, ultimately shaping the final membrane structure and performance (**Scheme** **1**).

## 2 Results and Discussion

### 2.1 Incipient Film Formation

**Figure 1:**
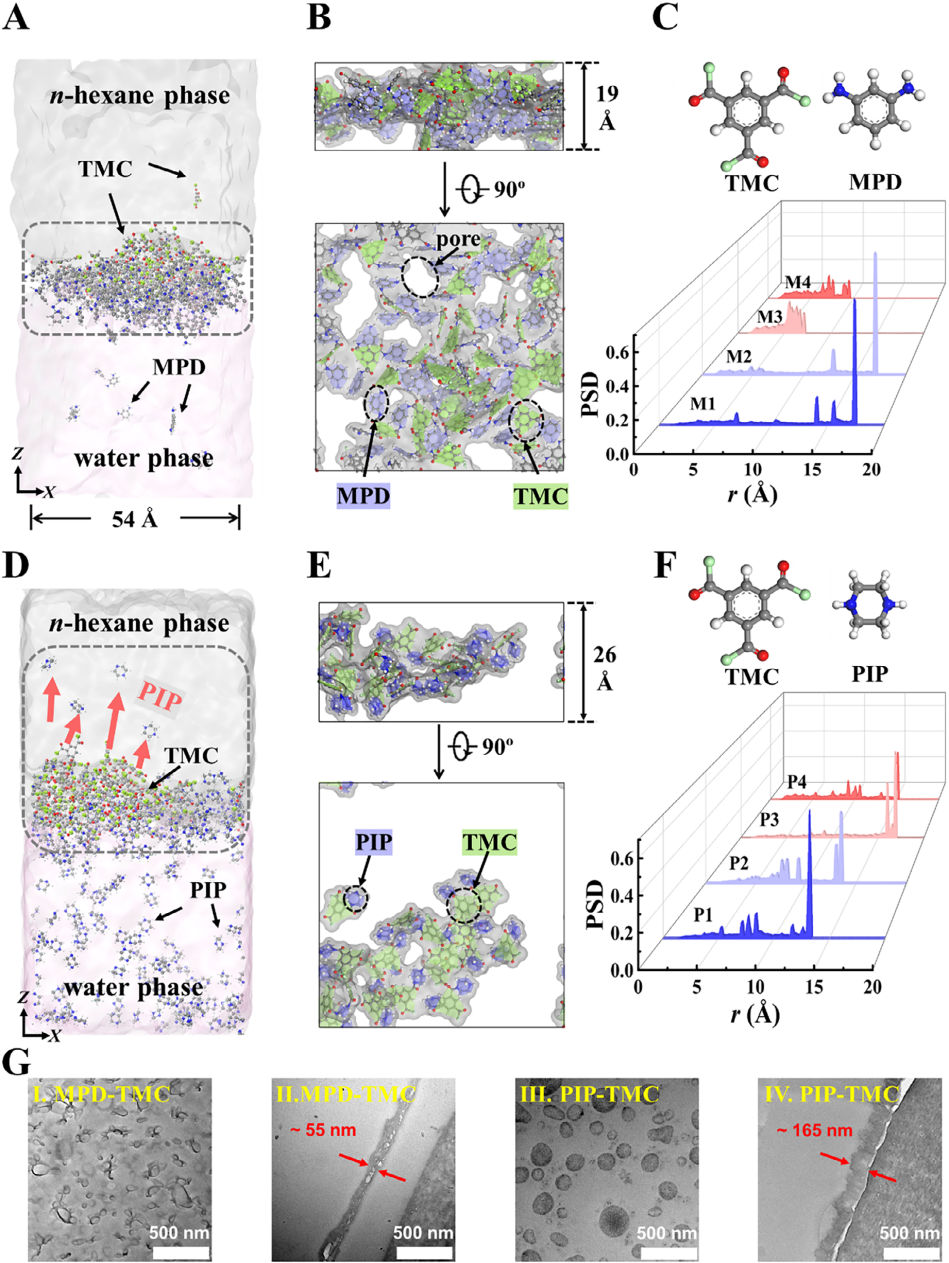
Formation of incipient polyamide films. A) Snapshot of the MPD‐TMC M3 system (Table S1, Supporting Information) after 5 ns of equilibrium diffusion. B) MPD‐TMC polyamide film after in situ crosslinking. Top: Cross‐sectional view. Bottom: Top‐down view. C) Pore size distribution (PSD) of incipient polyamide films formed in MPD‐TMC systems at varying concentrations (Table S1, Supporting Information). D) Snapshot of the PIP‐TMC P3 system (Table S2, Supporting Information) after 5 ns of equilibrium diffusion. E) PIP‐TMC polyamide film after in situ crosslinking. F) PSD of incipient polyamide films obtained in PIP‐TMC systems at varying concentrations (Table S2, Supporting Information). G) TEM images of the final polyamide membranes formed from MPD–TMC and PIP–TMC systems. I, III: surface; II, IV: cross‐section.

The neat water/*n*‐hexane interface features an 8.0 ± 1.0 Å depletion layer (Figure S1, Supporting Information), consistent with previous studies.^[^ ^29^, ^30^ ^]^ However, introducing MPD‐TMC or PIP‐TMC reactants eliminates this layer, as amine monomers migrate to the interface within 5 ns. MPD molecules accumulate uniformly in a narrow zone (**Figure** **1**A), while PIP monomers distribute across three domains—remaining in bulk water, accumulating at the interface, or penetrating the *n*‐hexane phase (Figure 1D). In contrast, the electrophilic TMC diffuses more slowly due to asymmetric solubility between the phases (Figure S4, Supporting Information).

Incipient film formation begins once reactants reach a quasi‐stationary concentration.^[^ ^31^ ^]^ At 5 ns post‐diffusion, in situ crosslinking (Figures S5 and S6, Supporting Information) reveals that the MPD‐TMC film is thin (≈19 Å), dense, with ≈5.3 Å pores (Figure 1B,C), while the PIP‐TMC film is thicker (≈26 Å) and looser, with pores exceeding 15 Å (Figure 1E,F). Despite PIP's higher reactivity with TMC (Figure S7 and Table S4, Supporting Information), its uneven distribution results in a less dense network. These properties align with experimental RO^[^ ^20^, ^32^ ^]^ and NF^[^ ^33^ ^]^ membrane characterizations (Figure S8, Supporting Information).

Furthermore, film thickness increases with monomer concentration and reaction time within the scope of this study, while free volume and pore size decrease (Figure 1C,F; Figures S9–S16, Supporting Information). Based on kinetic models, the incipient film—whether impermeable (MPD‐TMC) or permeable (PIP‐TMC)—seals the interface, limiting further monomer diffusion and driving diffusion‐limited polyamide growth, ultimately shaping the structure and morphology of the mature membrane. This results in a dense MPD‐TMC barrier within a looser RO membrane structure,^[^ ^28^ ^]^ reducing permeability but enhancing salt rejection compared to the more open NF membranes.

To support the observed differences in interfacial migration and incipient film formation between the MPD‐TMC and PIP‐TMC systems, we conducted transmission electron microscopy (TEM) on freestanding polyamide membranes formed after 2 min of IP (Figure 1G). The MPD‐TMC membrane exhibited a relatively thin and compact structure with a thickness of ≈55 nm, whereas the PIP‐TMC membrane was significantly thicker (≈165 nm), indicating a looser, more extended polymer network. These trends are also in agreement with previous AFM measurements at lower monomer concentrations (0.1 wt.%), where the thicknesses of freestanding MPD‐TMC and PIP‐TMC membranes were reported as ≈8.4 and ≈33.2 nm (Figure S8, Supporting Information), respectively.^[^ ^20^ ^]^ The morphological differences are consistent with our simulation results, which predict distinct water finger dynamics and monomer transport behaviors at the aqueous‐organic interface for each monomer system.

### 2.2 Water Fingers and Monomer Transport

The presence of water fingers at the interface alters the trajectory and final destination of monomer molecules (as illustrated in Scheme 1), ultimately influencing the morphology of the incipient polyamide film. When MPD molecules interact with the interface, they are frequently stabilized by transient water structures that extend into the organic phase, yet these fingers are relatively short‐lived and dissipate quickly. In contrast, PIP molecules are more deeply embedded within these water fingers, which are longer and more persistent, facilitating their transfer into the *n*‐hexane phase. The contrast in water finger lifetimes and penetration depth directly impacts how far and how fast monomers can move into the organic phase for subsequent polymerization.

**Figure 2:**
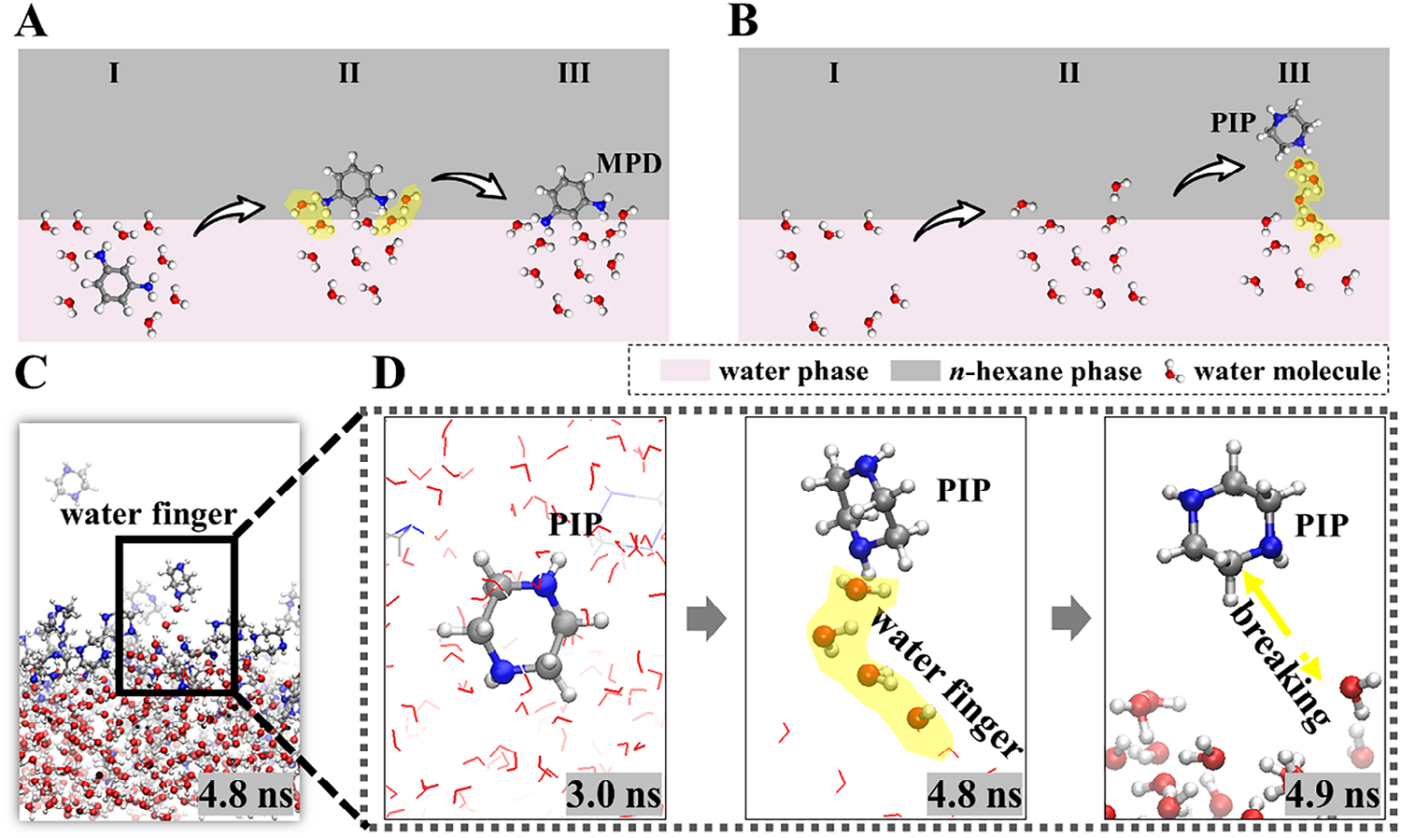
Water finger dynamics during trans‐interface migration of amine monomers. Water fingers (highlighted in yellow) are clusters of water molecules (<10 Å in length) that extend from the water phase into the *n*‐hexane phase. Schematic representation of water‐MPD A) and water‐PIP B) interactions during sub‐processes I (water to interface), II (interface), and III (interface to *n*‐hexane). C) Snapshot of interfacial water fingers in PIP‐TMC P3 system at 4.8 ns. D) Dynamic sequence illustrating the formation and breaking of a water finger.

A detailed examination of the trans‐interface migration of the two amine monomers, categorized into three sub‐processes—(I) water to interface, (II) interfacial transport, and (III) interface to *n*‐hexane—reveals distinct differences in their distribution within the IP reaction zone (**Figure** **2**A,B). The reduced local diffusion coefficient observed in sub‐process II (Figures S17 and S18 and Tables S5–S7, Supporting Information) coincides with the emergence of water fingers during sub‐processes II and III (Figure 2; Figure S19 and Movies S1 and S2, Supporting Information). The water fingers, originating from interfacial fluctuations caused by thermally excited capillary waves, have been proposed as a key mechanism of interfacial ion transport^[^ ^11^, ^12^ ^]^ and micro‐solvation.^[^ ^13^ ^]^ These fingers are tightly linked to the electrostatic potential distribution of solutes (Figure S20, Supporting Information), owing to the nucleophiles’ propensity to retain some water molecules as hydration shells upon approaching the interface. This coupling can impede solute motion relative to unhindered simple diffusion, as described in classical IP theories.^[^ ^16^ ^]^

**Figure 3:**
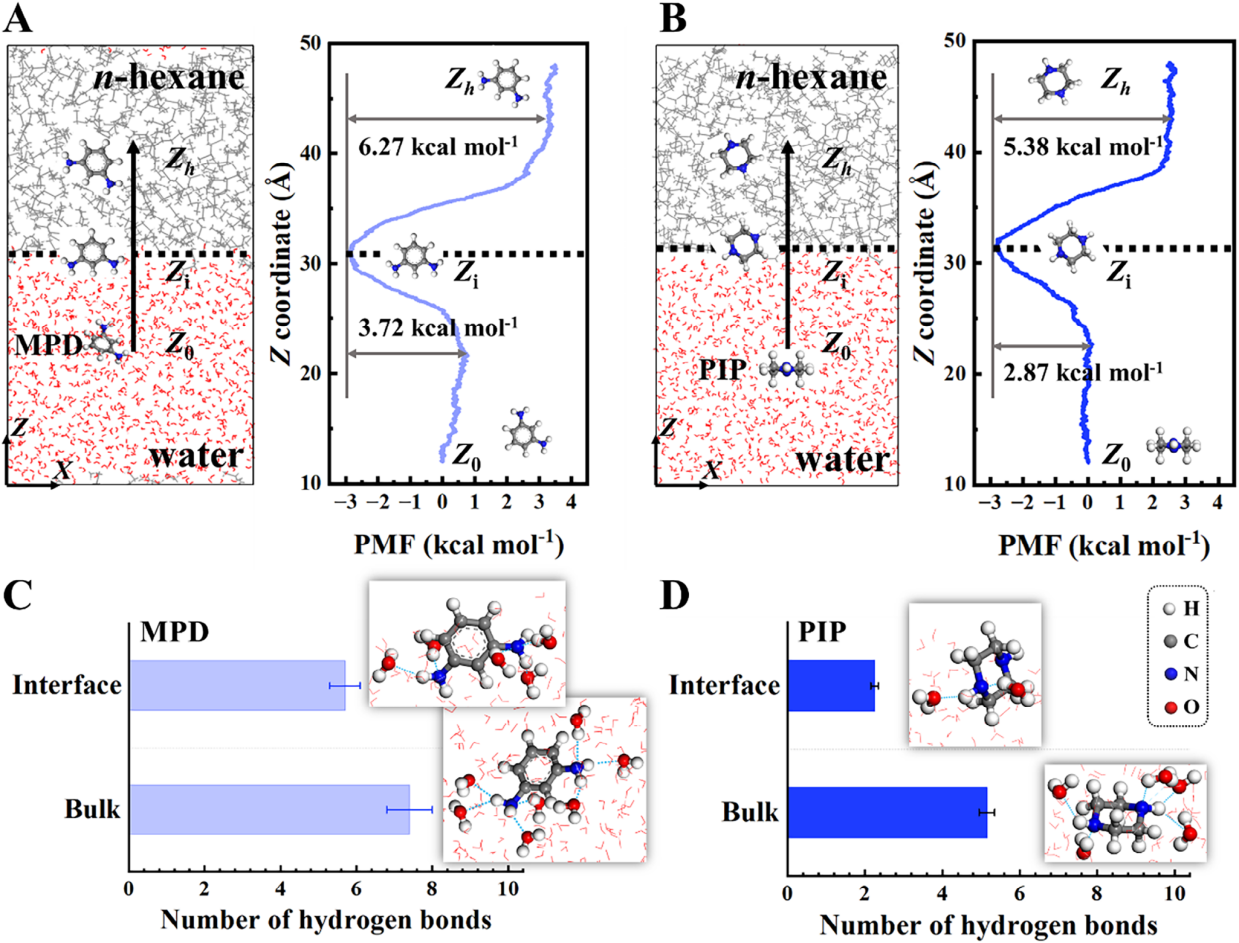
Driving force of water finger formation. PMF profiles for MPD A) and PIP B) molecules at different locations along the *Z*‐coordinate in the water/*n*‐hexane system. Dashed lines indicate the aqueous‐organic interfacial boundaries. Number of hydrogen bonds formed by MPD C) and PIP D) molecules at different *Z*‐coordinate locations.

Considering the entire process (≈5 ns), to identify the driving forces behind monomer transport from the aqueous phase to the *n*‐hexane phase, we determined the potential of the mean force (PMF) for individual monomers along the interface normal (*Z*‐axis) using umbrella sampling.^[^ ^34^ ^]^ As shown in **Figure** **3**A,B, the free energy of transfer from water to the interface (sub‐process I) was −3.72 kcal mol^−1^ for MPD and −2.87 kcal mol^−1^ for PIP (at 25 °C), favoring both but leading to a higher proportion of PIP remaining in water (Figure 1A,D). In addition, both MPD and PIP monomers exhibit energy minima at the water/*n*‐hexane interface, indicating a preference to reside in the interfacial region, where they experience the lowest free energy, signifying their surface activity.^[^ ^1^ ^]^

The energy barrier for transition from the interface to the *n*‐hexane phase (sub‐process III) at 25 °C is higher for MPD (6.27 kcal mol^−1^) than for PIP (5.38 kcal mol^−1^). For MPD, this barrier is ≈2.8 times the strength of a hydrogen bond in liquid water (2.25 kcal mol^−1^),^[^ ^29^ ^]^ rendering its partitioning into the *n*‐hexane phase less favorable.^[^ ^34^ ^]^ Consequently, MPD flux is more easily halted at the organic side of the reaction zone.

The PMF profiles are consistent with observed differences in water finger interactions with the two amine monomers MPD and PIP. Animated trajectories show that, for PIP, despite the presence of an energy barrier, water fingers—clusters of water molecules within ≈10 Å—act as precursors for interfacial transport, increasing the depth to which PIP molecules penetrate into the *n*‐hexane phase (Figure 2B; Movie S2, Supporting Information). Upon reaching a critical penetration depth, the PIP molecule strips off its associated water (Figure 2D). Once a water finger disconnects, the fingering effects dissipate as the PIP monomer moves further into the organic phase. The formation and breaking of these water fingers provide a pathway for the transport of PIP molecules across the interface.

In contrast, owing to a higher energy penalty (Figure 3A), MPD migration is hindered by the water fingers, confining MPD molecules to the interface. The water fingers interacting with MPD at the interface persist for shorter durations (50–100 ps) compared to those with PIP (50–300 ps), further limiting MPD's penetration depth into the *n*‐hexane phase (Movie S1 and Figure S19, Supporting Information). The facilitation of PIP transport by water fingers is consistent with its higher partition coefficient (7.0 × 10^−2^ for PIP vs 5.0 × 10^−3^ for MPD; Figure S4, Supporting Information), evidenced by partitioning experiments. This enhanced partitioning underscores the role of water fingers in modulating the interfacial behavior of monomers, ultimately shaping the properties of the resultant polymer films.

The differences observed in the PMF profiles and water fingers interactions between the two monomers can be attributed to the subtle variations in their molecular structures. MPD, with two hydrophilic amino groups directly attached to a rigid benzene ring (Figure S20, Supporting Information), exhibits stronger interactions with water (Figure 3C), resulting in shorter‐lived and shallower water fingers that restrict its penetration into the *n*‐hexane phase. PIP, bearing amino groups on a flexible piperazine ring, forms more persistent and deeply extended water fingers (Figure 2), promoting its deeper transport across the interface. The binding energies of MPD with water and *n*‐hexane are higher than those of PIP (Figure S21 and Table S8, Supporting Information). As a result, MPD molecules preferentially adopt a stable orientation where the two amino groups interact with the aqueous phase, while the benzene ring penetrates the organic phase (Figure 3A). This orientation renders MPD molecules less susceptible to water finger dynamics.

To quantify the strength of water fingers, we analyzed the average number of hydrogen bonds formed by each monomer in bulk and interfacial environments. MPD maintains a hydration shell of 7.4 water molecules in bulk water and retains 5.7 upon migration into the *n*‐hexane phase (Figure 3C). In contrast, PIP, which attracts 5.1 water molecules in bulk, retains only 2.2 at the interface (Figure 3D), reflecting its greater tendency to escape the hydrogen‐bonding network. Furthermore, interfacial water molecules exhibit reduced hydrogen bonding (2.3 bonds in the MPD system and 1.5 in the PIP system) compared to bulk water (averaging 3.4 bonds) (Figure S22, Supporting Information).

The orientation and hydrogen bonding behavior of amine monomers at the interface are closely tied to their molecular geometry. While this study focused on MPD and PIP, the impact of positional isomers such as *p*‐phenylenediamine (*p*‐PDA) and *o*‐phenylenediamine (*o*‐PDA) presents an intriguing avenue for further research (Figure S23, Supporting Information).

The dynamic behavior of water fingers challenges conventional IP theories, which have long described amine monomer transport as simple diffusion driven by a concentration gradient. This holds true only if the PMF profile decreases monotonically across the interface. However, observed energy barriers and water finger formation indicate that monomer transport is an activated process driven by these transient structures rather than passive diffusion.

### 2.3 Modulation of Water Fingers

We further show that polyamide network properties can be tuned by modulating water fingers (Figures S24–S33 and Movies S3–S7, Supporting Information).^[^ ^35^, ^36^ ^]^ Comparative analysis of MPD‐TMC and PIP‐TMC systems at equivalent monomer concentrations reveals that water fingers play a critical role in generating thicker and looser polyamide networks, especially in systems with higher monomer concentrations (Figures S24–S29 and Movies S3 and S4, Supporting Information). Generally, the crosslinking degree and density of the resulting incipient polyamide film increase with monomer concentration, while the average pore size and free volume fraction decrease (Figure S33, Supporting Information). Water fingers significantly influence these properties. In MPD‐TMC systems, water fingers exhibit greater activity in the M4 system than in the M3 system, resulting in a more porous structure characterized by higher free volume fractions and lower density in M4. Similar water finger effects are observed in PIP‐TMC systems, particularly across the P1 to P3 systems.

Additionally, water finger formation can be enhanced by introducing co‐solvents (Figure S30 and Movie S5, Supporting Information) or micellar surfactant solutions (Figure S31 and Movie S6, Supporting Information) into the liquid–liquid interface. These strategies induce interfacial instabilities that reorganize the hydrogen‐bonding network, thereby driving the development of water fingers. In the MPD‐TMC system, adding a surfactant such as sodium dodecyl sulfate (SDS) at concentrations exceeding its critical micelle concentration (CMC) disrupts the interfacial structure, disperses water fingers heterogeneously, and enhances MPD partitioning into the organic phase (Figure S31). This leads to membranes with rougher, looser surfaces and improved water permeability.^[^ ^35^ ^]^ Conversely, in the PIP‐TMC system, water fingers can be suppressed by introducing SDS solution at concentrations below its CMC (Figure S32 and Movie S7, Supporting Information). In this case, the SDS monolayer stabilizes and flattens the interface, yielding a denser, smoother polyamide network compared to conventional NF membranes. These modified membranes hold promise for separating multivalent ions with comparable sub‐nanoscale dimensions.^[^ ^36^ ^]^

The formation of water fingers at the water/*n*‐hexane interface is primarily driven by thermally induced capillary waves and local interfacial fluctuations. These dynamic protrusions are highly sensitive to system parameters such as temperature, interfacial tension, and interfacial curvature. Our findings highlight that water finger dynamics can be actively modulated through thermodynamic (e.g., temperature, (Figure S34, Supporting Information)) and chemical (e.g., interfacial tension modifiers, (Figure S30–32, Supporting Information)) means. Such tunability opens up new opportunities for controlling interfacial transport and tailoring membrane properties in IP systems. We acknowledge that interfacial curvature—especially in emulsions, vesicles, or confined geometries—can significantly affect water finger formation. Although our current simulations use a planar interface for clarity and control, curvature introduces local variations in Laplace pressure and capillary wave behavior, which may either promote or suppress finger formation depending on geometry and pressure gradients. Investigating these effects could broaden the applicability of our findings to more complex interfacial polymerization systems.

## 3 Conclusion

In summary, this work reveals that the interfacial migration of monomers during IP is not a simple diffusive process but one governed by transient, directional hydration structures. These “water fingers” act as nanoscale conduits that selectively modulate the transport of chemically distinct monomers, leading to vastly different polymer network structures. By resolving this mechanism, we provide a conceptual advance that unifies molecular orientation, interfacial energetics, and polymer morphology in IP. This insight broadens the foundational understanding of aqueous interfaces and enables the rational design of next‐generation membranes and polymeric nanomaterials through interfacial engineering.

Importantly, the “water finger” mechanism is likely generalizable beyond MPD–TMC and PIP–TMC systems. We propose that monomer properties such as rigidity, hydrophilicity, and hydrogen‐bonding capacity strongly influence their coupling with interfacial dynamics. This framework opens avenues for tuning transport behaviors via molecular design—extending to aliphatic, heterocyclic, or functionalized diamines—and optimizing membrane performance.

Looking forward, this atomistic perspective lays the groundwork for rationally controlling interfacial phenomena across chemistry, biology, and materials science by harnessing the principles of dynamic hydration and nanoscale transport.

## 4 Experimental Section

### Measurement of Oil/Water Partition Coefficients of Amine Monomers

An aqueous MPD solution (30 mL, 1.0 wt.%) was prepared and poured into a beaker (100 mL). Subsequently, 30 mL of *n*‐hexane solution was gently injected onto the surface of the aqueous solution. After 120 s, *n*‐hexane (≈3 mL) was collected from the air/*n*‐hexane interface and placed in a disposable cell (4.5 mL, 2‐478‐06, AS ONE Corporation, Japan). The absorbance variation of MPD (maximum absorption peak: ≈289 nm) in *n*‐hexane was detected by UV–vis spectroscopy (V‐650KE; JASCO Company, Japan). Subsequently, the variation in the absorbance of the MPD with time was analyzed using fixed‐wavelength UV–vis absorption spectra (wavelength: ≈289 nm). The as‐prepared aqueous solution (1 mL) was gently injected into a 4.5 mL disposable cell. The height of the aqueous solution (≈1 cm) was below the light path of the spectrometer. Subsequently, 1.5 mL of *n*‐hexane solution was slowly added to the surface of the aqueous phase within 15 s. The amount of MPD that diffused into the *n*‐hexane phase from 15 to 120 s was determined using UV–vis absorption spectroscopy, and calculated from the standard curve.^[^ ^35^ ^]^ The partition coefficient of MPD was calculated based on the ratio of the MPD concentrations in the organic and water phases. The solubility of MPD in *n*‐hexane phase was calculated by dissolving excessive MPD (100 mg) into the organic liquid (20 mL) and measured the UV–vis absorption spectroscopy of the supernatant solution, and then transferring to MPD concentration in *n*‐hexane.

The oil/water partition coefficient and solubility of PIP were obtained using the same method, except that the wavelength of the UV–vis absorption spectra was 254 nm.

### Fabrication of Freestanding Polyamide Nanofilm

Freestanding polyamide nanofilms of the MPD‐TMC and PIP‐TMC systems were fabricated using a support‐free IP method.^[^ ^35^ ^]^ An aqueous solution containing 0.25 wt.% MPD was prepared under stirring for 40 min and gently poured onto a glass slide, followed by the addition of *n*‐hexane solution containing 0.15 wt.% TMC. The two immiscible phases were allowed to come into contact for 2 min. Subsequently, the aqueous and organic phase solutions were immediately drained to obtain freestanding polyamide membranes, which were washed with DI water. Finally, the free‐standing polyamide membranes were transferred onto different support membranes and further post‐treated in a ventilated oven at a temperature of 60 °C for 5 min. For surface characterization, an anodic aluminum oxide isotropic (AAOI, Pore size 100 nm, Alliance Bio. Com., Japan) was used as the support membrane. For the cross‐sectional characterization, polyacrylonitrile (PAN) ultrafiltration membrane (50 kDa) was used as the support. Finally, the resulting support membranes were cooled to room temperature for further characterization.

### MD Simulations of Monomer Diffusion Across the Water‐Oil Interface

MD simulations to investigate the interfacial properties during the diffusion of monomers across the interface were performed based on the quasi‐steady‐state approximation using the Forcite module with the condensed‐phase optimized molecular potential for atomistic simulation study III (COMPASS III) force field in the commercial software BIOVIA Materials Studio 2023.^[^ ^35^ ^]^ The compositions of the water/*n*‐hexane interface, MPD‐TMC, and PIP‐TMC systems are listed in Tables S1 and S2 (Supporting Information). Notably, the concentrations of the amine and TMC monomers were higher than the experimental values because of limited simulation volumes. For each system, a simulation box with periodic boundary conditions applied in all 3D was built in a rectangular box, and the interface was parallel to the *XY* plane (*X = Y =* 52–58 Å, depending on the composition of the system). Vacuum slabs above the *n*‐hexane phase and under the water phase were introduced to preclude interactions between the periodic images of *n*‐hexane and water molecules (Figures S1–S3, Supporting Information). By appropriately setting the positions of all the molecules, a geometry optimization process was first performed, followed by a dynamic run for 2 ns with the NVE ensemble (Table S3, Supporting Information). The Ewald summation method was used for electrostatic interactions. To ensure that all systems reached equilibrium, another run of 5 ns with the NVT ensemble was applied. The temperature was maintained at 298 K by using a Nose thermostat with a Q ratio of 0.01. After 5 ns, the energy and temperature profiles of all systems reached steady values. The animated trajectories of the final 5 ns run in the NVT were used for data analysis. All the systems were run twice to ensure the accuracy of the results.

### Potential of Mean Force (PMF)

The free energy of the amine monomers (MPD and PIP) required to overcome the trans‐interface resistance from the water phase to the *n*‐hexane phase was calculated in the form of the PMF by a combination of umbrella sampling and weighted histogram analysis.^[^ ^34^ ^]^ For each amine monomer, a simulation cuboid (dimensions: *X* = *Y* = 32 Å, Z = 60 Å) composed of 1043 molecules of water in the bottom layer and 234 molecules of *n*‐hexane in the top layer was constructed. The sampling path for the PMF calculation was defined by pulling the center of mass of the amine monomer from *Z* = 12 Å in the center of the water phase as the initial position to *Z* = 48 Å in the *n*‐hexane phase as the final position. The force constant of the umbrella potential used in the sampling was set to 6 kcal mol^−1^ Å^−2^. Reaction coordinates were separated into 36 windows within the total distance between the initial and final positions. Each sampling window was run in NVT at 298 K for 10 00 000 equilibration steps and subsequently for 50 00 000 production steps, with a time step of 1 fs. The weight histogram analysis method was used to calculate the free energy. All the PMF profiles were normalized to zero at the initial position (*Z* = 0).

## Conflict of Interest

The authors declare no conflict of interest.

## Supporting information



Supporting Information

Supplemental Movie 1

Supplemental Movie 2

Supplemental Movie 3

Supplemental Movie 4

Supplemental Movie 5

Supplemental Movie 6

Supplemental Movie 7

## Data Availability

The data that support the findings of this study are available in the supplementary material of this article.
