# Supplementary material for: Dynamic Sub‐Nanoscale “Water Fingers” in Interfacial Polymerization
Source: Small. 2025 Jun 20;21(33):2504497. doi: 10.1002/smll.202504497 (PMC12372459; doi:10.1002/smll.202504497)
Supplement: Supplementary file 1 — Supporting Information [file SMLL-21-2504497-s008.docx]

Supporting Information

Dynamic sub-nanoscale “water fingers” in interfacial polymerization

Zhaohuan Mai, Tomohisa Yoshioka, Akshay Deshmukh, Tianmu Yuan, Junyong Zhu, Jinkai Yuan, Ralph Rolly Gonzales, Ayano Yamamoto, Yongxuan Shi, Wenming Fu, Kecheng Guan, Zhan Li, Pengfei Zhang, John H Lienhard, Hideto Matsuyama*

Dr. Z. Mai, Prof. T. Yoshioka, Dr. R. R. Gonzales, Dr. Y. Shi, Dr. W. Fu, Dr. K. Guan, Dr. Z. Li, Dr. P. Zhang, Prof. H. Matsuyama*

Research Center for Membrane and Film Technology, Kobe University, Kobe, 657-8501, Japan

E-mail: [matuyama@kobe-u.ac.jp](mailto:matuyama@kobe-u.ac.jp)

Prof. T. Yoshioka, A. Yamamoto

Graduate School of Science, Technology and Innovation, Kobe University, Kobe, 657-8501, Japan

Dr. A. Deshmukh

Department of Mechanical Engineering, Massachusetts Institute of Technology, Cambridge, MA 02139-4307, USA

Dr. T. Yuan

Department of Chemical Engineering, The University of Manchester, Manchester, M1 3AL, UK

Prof. J. Zhu

School of Chemical Engineering, Zhengzhou University, Zhengzhou, 450001, PR China

Prof. J. Yuan

Laboratoire de Chimie de la Matière Condensée de Paris (LCMCP), Sorbonne Université, CNRS, UMR 7574, Paris, 75005, France

Dr. Y. Shi, Dr. W. Fu, Prof. H. Matsuyama*

Department of Chemical Science and Engineering, Kobe University, Kobe, 657-8501, Japan

**The PDF file includes:**

Theories

Computational simulations

Experimental materials and methods

Supplementary Text

Figs. S1 to S34

Tables S1 to S8

References

**Other Supplementary Materials for this manuscript include the following:**

Movies S1 to S7

**1. Theories**

**1.1 Liquid-liquid interface**

The counterintuitive anomalies observed in the physicochemical properties of liquid water can be attributed to its dynamic three-dimensional hydrogen-bonding network, which depends on the delicate interplay between the local interactions and long-range electrostatic interactions of water molecules (*14*, *15*). This well-defined network (especially in the outermost layer) would undergo dynamic rearrangement when the liquid water comes into contact with a second phase (i.e., vapor, another immiscible liquid, or a solid), as substantial evidence from both experimental measurements and molecular dynamics (MD) simulations has indicated the formation of a “depletion layer” accompanied by an asymmetric hydrogen bond distribution at the interface between water and a hydrophobic surface, with an interfacial width in the order of a few Ångströms to nanometers (*4*–*10*). In such a confined environment, thermal fluctuations usually generate a rough surface at the nanoscale as described by the capillary wave theory (*4*). These characteristic structural fluctuations are critical to the behavior of solutes at interfaces (*11*).

**1.2 Kinetics of interfacial polymerization (IP)**

A distinct challenge in the specific investigation of the water-oil interface structure during the IP process lies in the capabilities of both experiments and simulations, as the formation of ultrathin films (10–100 nm) by IP is a rapid diffusion–reaction process that is far from thermodynamic equilibrium. In realistic experimental scenarios, *m*-phenylene diamine (MPD) and piperazine (PIP) molecules are consumed by the reaction with trimesoyl chloride (TMC) in the reaction zone and supplied continuously from the aqueous phase during the experimental IP process on a timescale of a few seconds to minutes.

Typical IP processes can be divided into three stages (*28*). After contact between the two immiscible phases, MPD (or PIP) molecules diffuse from the water phase into the *n*-hexane phase (or at least into the interfacial zone near the *n*-hexane phase) and immediately react with the TMC monomers (Stage 1). This is because of the better solubility of MPD (or PIP) in the organic phase than that of TMC in the water phase. The incipient film is formed within a finite reaction zone (perhaps ~ nanometer) during the initial period and is considered the densest of the final mature polyamide network. This initial polyamide film is likely to seal the interface and largely inhibit subsequent monomer diffusion and IP reactions, resulting in the diffusion-limited growth of polyamide fragments in Stage 2. The IP process is self-terminated, and the final mature polyamide membrane is formed in Stage 3, when there is no more MPD (or PIP) diffusing into the organic phase. The basic conclusion in membrane area is that the incipient film formed at the initial stage is of great importance in determining the structure and performance of the final thin film composite membrane.

Because MPD (or PIP) has a low partition coefficient from water into *n*-hexane, there is a high diffusion energy barrier for the transport of MPD (or PIP) into *n*-hexane. Consequently, only MPD (or PIP) molecules with energies larger than the energy required to overcome the diffusion energy barrier can be transported across the water/*n*-hexane interface and react with TMC to form the polyamide fragment. The diffusion of the MPD (or PIP) tends to be random at different locations. In addition, the heat released from the reaction between MPD (or PIP) and TMC may provide energy to MPD (or PIP) molecules in the vicinity and induce faster diffusion of MPD (or PIP) around this area. Conversely, the locations where successful diffusion of MPD (or PIP) did not occur remain less favorable for diffusion across the water/*n*-hexane interface. The heterogeneous diffusion of MPD (or PIP) across the water/*n*-hexane interface possibly generates a series of reaction sites with different crosslinking degrees, that is, locations with abundant and insufficient reactive monomers constitute high and low crosslinking degree spots for the polyamide fragments, respectively. This is applicable to both Stages 1 and 2. The only difference is that in Stage 2, an incipient polyamide film is already formed at the interface, which is also a barrier preventing MPD (or PIP) from diffusing through it and continuing to react with TMC. As the polyamide fragments formed in Stage 2 continue to grow, they eventually connect and form a dense polyamide layer with a large distribution of crosslinking degrees and a broad distribution of pore sizes (Stage 3). Therefore, the heterogeneous growth rate of polyamides at different locations in *n*-hexane leads to a final polyamide membrane with a non-uniform and relatively broad pore size distribution compared to the incipient film.

Two key points should be emphasized: (1) the structure of the incipient polyamide film (Stage 1), which ultimately determines the structure and morphology of the final mature polyamide membrane (Stage 3), and (2) the slow and non-uniform trans-interface diffusion of MPD (or PIP) in Stage 2, which is difficult to access through experimental characterizations and computational simulations.

**1.3 Molecular dynamics (MD) simulations of IP**

Computer simulations, particularly MD simulations, have become valuable tools in material science in recent decades. The progress in MD simulations and coarse-grained models for polyamide membranes has been reviewed in a previous study (*37*). The corresponding time and length scales for different simulation methods are shown in Fig. S7 in (*35*) compared with the time and length scales of polyamide membrane formation process. However, it is worth noting that current computational resources are insufficient to simulate the polymerization process in atomic detail at realistic system sizes and experimental timescales. As MD simulations often, struggle to reproduce experimentally accessible dimensions in time and length, for instance, the complete IP reaction takes place on a timescale of a few seconds to minutes, whereas the system has an extremely broad range of timescales spanning many orders of magnitude. Considerable activity occurs on a range of timescales before the final polyamide membrane is formed: (1) monomer diffusion and incipient film formation (picoseconds to milliseconds), (2) diffusion-limited growth of the polymer network (milliseconds to seconds), and (3) self-limited growth of the final mature membrane (seconds to minutes). Whereas the basic time step of atomically detailed simulations is approximately femtoseconds to maintain the stability of the integration, processes that require seconds or longer, such as the diffusion-reaction IP process, are beyond the reach of current computational resources. The number of simulation steps (10^16^ steps to reach timescale of tens of seconds) is currently unattainable using available computers. Consequently, only a few studies have focused on simulating IP processes. However, despite these limitations, the MD simulations are suitable for investigating the initial period of the IP process. We anticipate that the simulation of submillisecond phenomena will become more accessible in the next few years, and the simulation of microsecond processes will become routine.

Nonetheless, although the MD simulations in this study can only provide information on the very beginning of the IP process regarding incipient film formation, they are still useful for predicting the IP characteristics in the subsequent stages and the properties of the final polyamide membrane.

**1.4 Quasi-steady-state approximation of monomer diffusion**

Quasi-steady-state approximation is a popular technique in chemical kinetics. This methodology relies on the assumption that the concentrations of all reactive intermediates experience negligible rates of change. This approximation is particularly reliable when the concentrations of intermediates are low as variables with small time derivatives tend to have small values if they do not exhibit rapid oscillations.

The reaction between the amine and TMC monomers can only commence once the amine monomers have successfully migrated from the water phase into the *n*-hexane phase (or at least in the interfacial zone near the *n*-hexane phase). We anticipate that the polymerization between MPD-TMC and/or PIP-TMC occurs on a characteristic time scale significantly later than the time required to achieve steady-state diffusion. Therefore, monomer diffusion was analyzed separately from the reaction process and addressed using the quasi-steady-state approximation method, which assumes that no reaction occurs before a certain number of amine molecules were transferred to the *n*-hexane side of the interface.

Furthermore, the IP reaction between MPD (or PIP) and TMC is extremely rapid under ideal conditions. Therefore, the polyamide membrane polymer networks produced may never reach true equilibrium, but instead quickly gel into an inhomogeneous and thermodynamically unstable configuration. However, in models of such materials, it is typically assumed that the actual membranes exist in a completely relaxed state at a given temperature-pressure ensemble (*37*).

**2. Computational simulations**

**2.1 MD simulations of monomer diffusion across the water-oil interface**

The MD model for the co-solvent assisted MPD-TMC system was constructed by adding 100 molecules of acetone in the *n*-hexane phase, and the numbers of other molecules were the same with the composition of M2 system. The dynamic diffusion process was conducted under the same conditions with M2 system.

The MD model for the micellar surfactant assisted MPD-TMC system was constructed by adding a monolayer comprised of 36 uniformly distributed sodium dodecyl sulfate (SDS) molecules placed at the water/*n*-hexane interface and an SDS micelle containing 50 SDS molecules with a radius of 15 Å underneath the SDS monolayer with a distance of 25 Å. The numbers of other molecules were the same with the composition of M2 system. The dynamic diffusion process was conducted under the same conditions with M2 system.

The MD model for the monolayer surfactant assisted PIP-TMC system was constructed by adding a monolayer comprised of 36 uniformly distributed SDS molecules placed at the water/*n*-hexane interface. The numbers of other molecules were the same with the composition of P3 system. The dynamic diffusion process was conducted under the same conditions with P3 system.

The snapshot of the MD simulation was produced by Materials Studio Visualizer and VMD software.

**2.2 MD simulations of monomer diffusion in the single solvent systems**

The diffusion properties of MPD and PIP in a single-solvent system were analyzed by constructing solutions in a single solvent. The MD models for the MPD-water and PIP-water systems comprised one MPD (or PIP) molecule and 200 water molecules. For the MPD/*n*-hexane and PIP/*n*-hexane systems, one molecule of MPD (or PIP) was added to 500 *n*-hexane molecules. The systems were geometry optimized, followed by an equilibration run in NPT at 298 K for 1 ns and a production run in NVT at 298 K for 5 ns.

**2.3 Diffusion coefficients of amine monomers**

The diffusion coefficients (*D*, m^2^ s^-1^) of MPD and PIP molecules in different MD systems described in **Sections 2.1** and **2.2** were analyzed from the slope of the mean square displacement (*MSD*) curves using the Einstein relationship (*35*):

| $MSD= \frac{1}{N}\sum_{1}^{N} \{[r (t)-r (0)]^{2}\}$ | | (S1) |
| --- | --- | --- |
| $D= \frac{1}{6}\lim_{N\to\infty} \frac{d}{dx} \{[r \left( t \right)-r \left( 0 \right)]^{2}\}$ | (S2) | |

where *N* is the total number of targeted molecules (i.e., MPD or PIP in this study), *r* (0), and *r* (t) represent the initial position (m) and the position at *t* (m) of the target molecule, respectively.

**2.4 Binding energy with solvent**

The binding energies of each amine monomer to the solvents were calculated using the equilibrium systems described in **Section 2.2**.

$E_{m-s}=E_{total}- E_{m}- E_{s}$ (S3)

where *E*_m-s_ (kcal mol^-1^) is the binding energy of the amine monomer with the solvent, *E*_m_ (kcal mol^-1^) is the energy of the amine monomer, and *E*_s_ (kcal mol^-1^) is the energy of the solvent.

**2.5 *In-situ* crosslinking**

After the dynamic diffusion process of the MPD-TMC and PIP-TMC systems described in **Section 2.1**, the interfacial zone of each system was extracted to conduct *in-situ* crosslinking, including all the solvent molecules and the two reactive monomers in the respective phase, as shown in **Fig. S5**. Compared to previous crosslinking protocols with no explicit solvent molecules (*39*), *in-situ* crosslinking is more realistic. Polymerization was defined based on atom-atom distance criteria, that is, when the distance between the C atom of the O=C-Cl group in one TMC molecule and the N atom of the NH_2_ group in one MPD (NH groups in PIP) molecule was < 5 Å (*38*). Consequently, the previously connected Cl atom from the acyl chloride group and H atom(s) from the amine group were replaced by C—N bonds. Unreacted TMC and MPD (or PIP) molecules were removed from the simulation zone. Then, the cross-linked network with water and *n*-hexane was relaxed by geometry optimization to redefine the new bonds, angles, and dihedrals. Finally, the two solvents were removed from the simulation cell, leaving only the crosslinked polyamide network. This network was considered the incipient film formed after the 5 ns course of equilibrium diffusion.

The degree of crosslinking (DC) of the polyamide network in each MD system was calculated using Eq. S4 as the ratio between the number of actual amide bonds and the theoretical maximum number of amide bonds if the polymer is fully cross-linked.

$DC=\frac{n_{C-N}}{n_{C-N}^{0}}=\frac{n_{N} - n_{NH2}}{n_{N}+ n_{C=O}}$ (S4)

where *n*_C-N_ is the number of amide bonds formed, calculated from the number of nitrogen atoms *n*_N_ minus the number of unreacted amine groups *n*_NH2_. The theoretical number of amide bonds, *N^0^*_C-N_, is the sum of all amine group numbers, *n*_N_ and the acyl chloride group number, *n*_C=O_.

The density, accessible free volume of water molecules, and pore size distribution (PSD) of the polyamide network in each system were analyzed using PoreBlazer (*39*) with a spherical probe with a radius of 1.38 Å.

For comparison, a conventional crosslinking procedure was also applied to both MPD- TMC and PIP- TMC systems without solvent molecules, following a previous MD study (*39*). 200 TMC and 300 MPD molecules (300 PIP molecules in PIP- TMC system) were randomly filled into a 3D-periodic box with lateral dimensions of 5 nm × 5 nm. Therefore, 600 potential reaction sites were available for crosslinking. The initial configuration was subjected to geometry optimization and a dynamic run of 5 ns in NVT at 298 K. After achieving equilibrium, crosslinking reactions were conducted using an initial cutoff distance of 3 Å between the reactive N and C atoms from the reactants. When all amide bonds available at the current cutoff distance were formed, the cross-linked polyamide network was further relaxed for 500 ps to optimize the configuration under the NPT ensemble. Such a crosslinking process continued with a stepwise increase in the cutoff distance of 0.5 Å until the cutoff distance reached 5 Å or the DC reached a constant value. Structural optimization with an automatically updated force-field assignment and 40 annealing cycles were performed for each cutoff distance until the final polyamide network was formed. Once the crosslinking reaction ceased, the ultimate DC was calculated using the method described in Eq. S4. The PSD profiles of the generated polyamide polymers generated by the non-*in situ* crosslinking procedure were compared with those obtained by *in-situ* crosslinking.

**2.6 Electrostatic potential (ESP)**

Quantum calculations were performed using the DMol3 module of the BIOVIA Materials Studio 2023 commercial software. The molecular structures of water, *n*-hexane, MPD, PIP, and TMC were optimized via Density Functional Theory (DFT) simulations using a generalized gradient approximation (GGA) with the PBE functional method. The *d*-type polarization function (DNP) basis set with the default SCF and geometrical convergence criteria was utilized for all geometric optimizations. The ESP isosurface of each molecule was determined by importing the electron densities and potentials into each molecule during the DFT calculations. The ESP – mapped van der Waals surfaces were plotted with the isosurface of the electron density gradient difference set to 0.005 a.u.

**2.7 Monomer reactivity**

To compare the reactivities of the MPD and PIP molecules with that of the TMC monomer, the Frontier Molecular Orbital (FMO) properties of the MPD, PIP, and TMC molecules were calculated to identify the population of the Highest Occupied Molecular Orbital (HOMO) and the Lowest Unoccupied Molecular Orbital (LUMO) in each molecule. All DFT simulations were performed using the DMol3 module in BIOVIA Materials Studio 2023 at the same level of theory described in **Section 2.6**. During molecular interactions (i.e., between MPD and TMC or between PIP and TMC), the LUMO accepts electrons and its energy corresponds to the electron affinity, whereas the HOMO represents electron donors and its energy is associated with the ionization potential. Therefore, the HOMO of nucleophilic MPD and PIP and the LUMO of electrophilic TMC were used to indicate their reactivity.

**2.8 Free energy of chemical reaction (Reaction energy)**

The Gibbs free energies of the reactions between MPD and TMC and between PIP and TMC were calculated via DFT simulations performed with the DMol3 module in BIOVIA Materials Studio 2023 at the same level of theory as described in **Section 2.6**. For each reaction, the DMol3 total energy yields the total electronic energy at 0 K. The results of vibrational analysis can be used to obtain important thermodynamic properties such as enthalpy (*H*), entropy (*S*), free energy (*G*), and heat capacity at constant pressure as functions of temperature. By combining the finite temperature corrections for the standard thermodynamic quantities, the free energy of each reaction, ${\Delta G}_{reaction}^{298.15K}$, can be calculated as follows (*19*):

$E_{T-corr}^{298.15K}=E_{Total}+ G_{Total}$ (S5)

${\Delta G}_{reaction}^{298.15K}=E_{T-corr}^{298.15K}\left( products \right)-E_{T-corr}^{298.15K}\left( reactants \right)$

$=E_{T-corr}^{298.15K}\left( polyamide \right)+E_{T-corr}^{298.15K}\left( HCl \right)-E_{T-corr}^{298.15K}\left( amine \right)- E_{T-corr}^{298.15K}\left( TMC \right)$ (S6)

where $E_{T-corr}^{298.15K}$ (kcal mol^-1^) is the total electronic energy of each component of the reaction after temperature corrections, calculated from the total electronic energy of this component at 0 K, $E_{Total}$(kcal mol^-1^), and the finite-temperature corrections for the free energy, $G_{Total}$ (kcal mol^-1^). The total electronic energies of all products at 298.15 K, $E_{T-corr}^{298.15K}\left( products \right)$, includes both polyamide fragment $E_{T-corr}^{298.15K}\left( polyamide \right)$ and the hydrogen chloride $E_{T-corr}^{298.15K}\left( HCl \right)$. In addition, the total electronic energies of all reactants at 298.15 K, $E_{T-corr}^{298.15K}\left( reactants \right)$, includes both anime monomer (MPD or PIP) $E_{T-corr}^{298.15K}\left( amine \right)$ and the TMC $E_{T-corr}^{298.15K}\left( TMC \right)$.

**2.9 Number of hydrogen bonding**

The number of hydrogen bonds between an amine monomer and its surrounding water molecules was calculated using the configurations generated in **Section 2.9** when the amine monomer transferred from the water phase to the organic phase. By explicitly defining the hydrogen acceptor and donor, the bond length, and the angle, the average number of hydrogen bonds in different zones can be obtained using the MD trajectories. The same method was used to calculate the number of hydrogen bonds among the water molecules.

**3. Experimental materials and methods**

**3.1 Experimental materials**

Trimesoyl chloride (TMC, > 98.0%), anhydrous piperazine (PIP, > 99.0%), and 1, 3-phenylene diamine (MPD, 99.0%) were purchased from Sigma-Aldrich, USA. Anhydrous *n*-hexane (99.0% purity) was purchased from Tokyo Chemical Industry, Japan.

**3.2 Experimental methods**

*Characterization of freestanding polyamide nanofilm:* The surface and the cross-sectional morphologies of the freestanding polyamide membranes were characterized by Transmission electron microscopy (TEM; JEM 2100 F, JEOL, Japan). All samples for TEM measurements were first entrapped in resin and then allowed to solidify at 60 °C for 2 days. The solidified samples were cut into 100 nm-thickness slides using a microtome (Ultramicrotome, Leica EM UC7, Germany).

**Supplementary Tex****t**


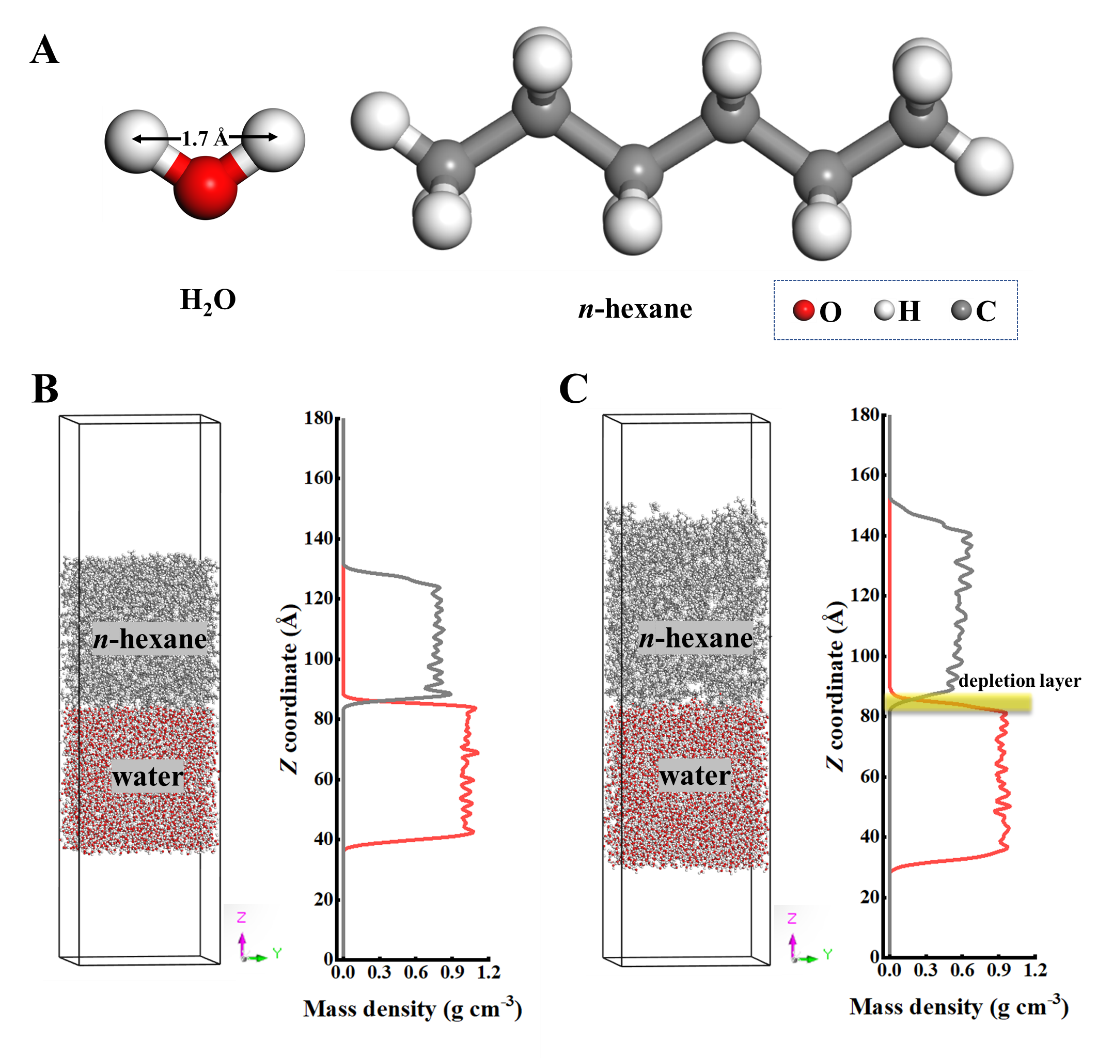


**Fig. S1. MD model of** **the water/*n*-hexane interface.** (A) Molecular structures of water and *n*-hexane. (B) Initial MD model of the water/*n*-hexane interface (left) with the mass density distribution as a function of the Z coordinate perpendicular to the interface. (C) Equilibrium MD snapshot of the water/*n*-hexane interface (right) with the mass density distribution as a function of the Z coordinate perpendicular to the interface.

A depletion layer was observed at the interface of the water/*n*-hexane system after equilibrium in the MD simulations during a period of 5 ns in NVT ensemble. However, unambiguous characterization of the width of the interface separating the two immiscible liquids is not possible. Following a previous work (*29*), we defined the width of the interface according to the density profiles obtained from the MD simulations. The depletion layer included the regions where the water density dropped from 90% to 10% of its bulk value and the *n*-hexane density increased from 10% to 90% of its bulk value. On this basis, the interfacial width was calculated to be 8.0 ± 1.0 Å in this study. From the plot of the interfacial width as a function of the simulation time, the depletion layer was established within the first 50 ps of the simulation and subsequently it was stable over the time period of the simulation.


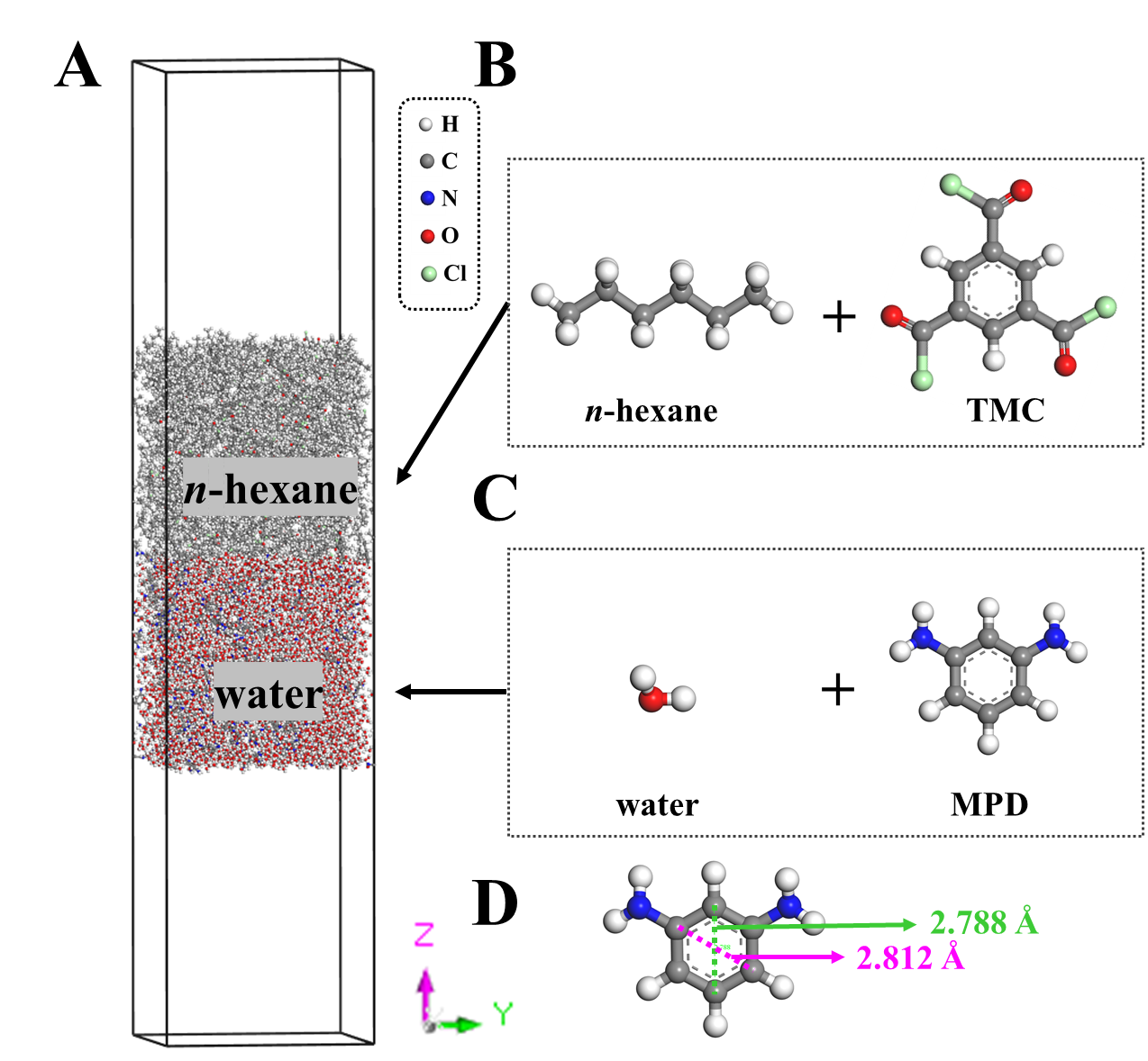


**Fig. S2. MD model of the water/*n*-hexane interface in MPD – TMC system.** (A) Snapshot of the initial MD model of the water/*n*-hexane interface in MPD – TMC system. (B) Molecular structures of *n*-hexane and TMC monomer in the organic phase. (C) Molecular structures of water and MPD monomer in the aqueous phase. (D) Dimensions of the MPD molecule.


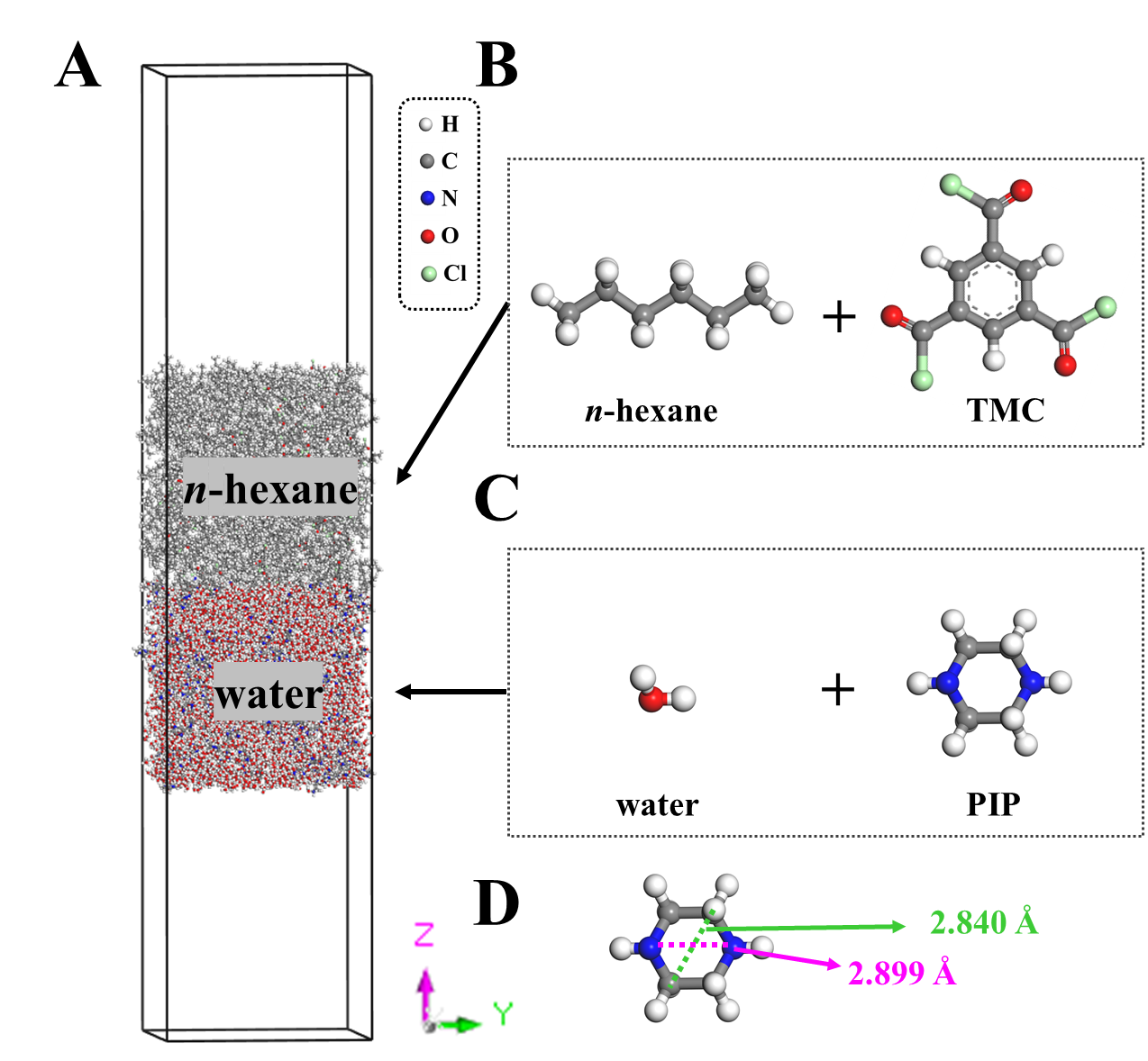


**Fig. S3. MD model of the water/*n*-hexane interface in PIP – TMC system.** (A) Snapshot of the initial MD model of the water/*n*-hexane interface in PIP–TMC system. (B) Molecular structures of *n*-hexane and TMC monomer in the organic phase. (C) Molecular structures of water and PIP monomer in the aqueous phase. (D) Dimensions of the PIP molecule.

**
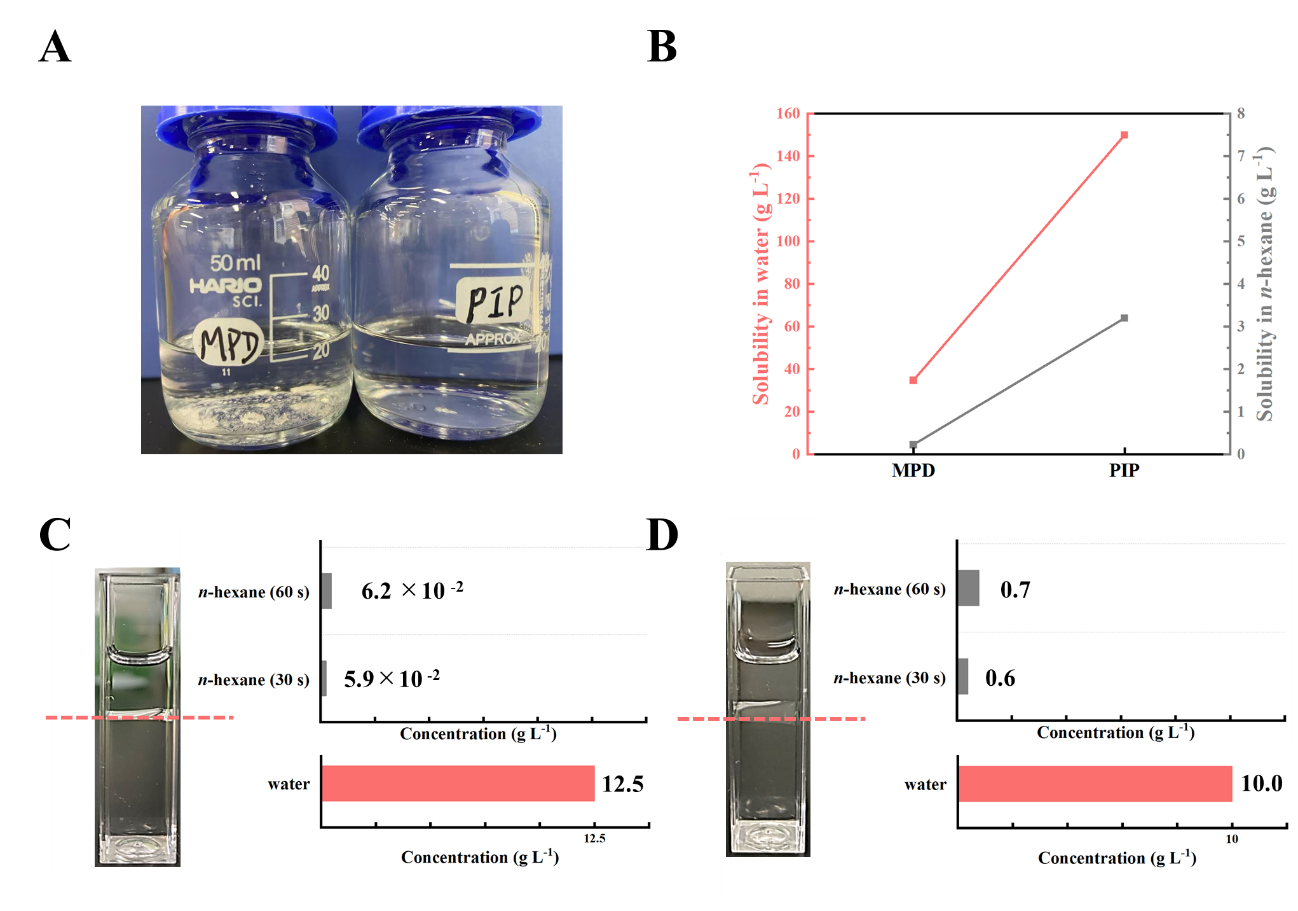
**

**Fig. S4. Measurement of *n*-hexane/water partition coefficients.** (A) Dissolution of amine monomers in *n*-hexane after 90 min. Left: MPD; right: PIP. (B) Comparison of monomer solubilities in water and *n*-hexane. (C) Partition coefficient of MPD calculated from concentrations in water and in *n*-hexane after different diffusion durations (30s and 60s). (D) Partition coefficient of PIP calculated from concentrations in water and in *n*-hexane after different diffusion durations (30s and 60s).

The two amine monomers (100 mg) were added in the *n*-hexane phase (20 mL) and agitated for 90 min. However, most of the MPD did not dissolve, and no apparent PIP particles could be visualized in *n*-hexane (Fig. S4 A), indicating a higher solubility of PIP than MPD in the *n*-hexane phase. From the standard curves, the measured solubilities of both amine monomers in the two solvents are shown in Fig. S4 B. For MPD, the saturated solubilities in water and in *n*-hexane are 38.4 and 0.23 g L^-1^, respectively. For PIP, the solubilities in both solvents are higher (150.0 g L^-1^ in water and 3.2 g L^-1^ in *n*-hexane, respectively). Fig. S4 C shows initial MPD concentration in the water and equilibrium MPD concentration in the oil after 30 s and 60 s of diffusion, indicating a partition coefficient *K*_MPD_ = 4.7 × 10 ^-3^ after 30 s diffusion from the water phase and *K*_MPD_ = 5.0 × 10 ^-3^ after 60 s diffusion. Using the same method, the partition coefficient of PIP was obtained as *K*_PIP_ = 6.4 × 10 ^-2^ (30 s diffusion) and 7.0 × 10 ^-2^ (60 s diffusion). These data were in the same order of magnitude with the those reported previously (*25*).


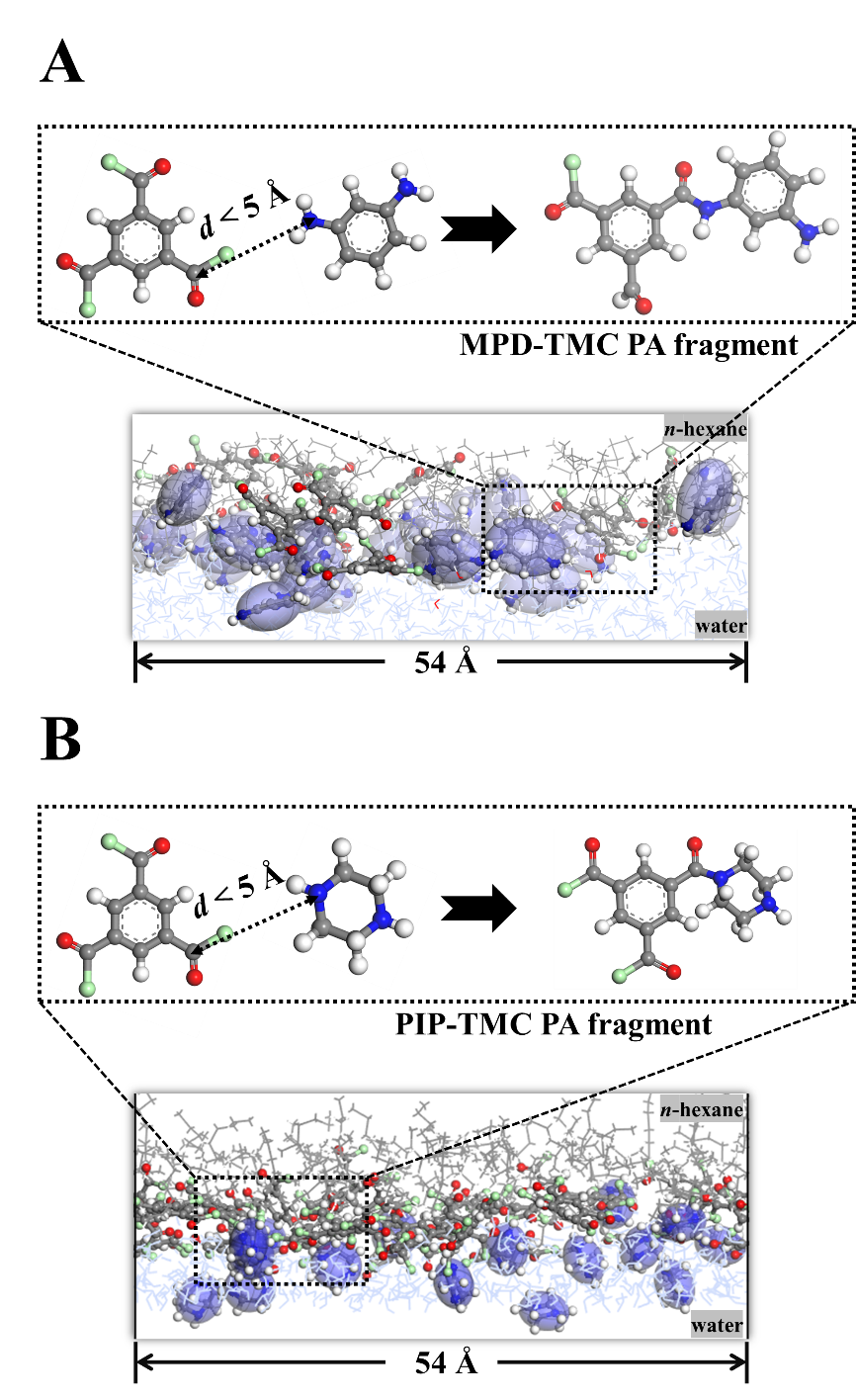


**Fig. S5. *In-situ* crosslinking procedures of the polyamide (PA) fragments.** (A) MPD-TMC system; (B) PIP-TMC system.


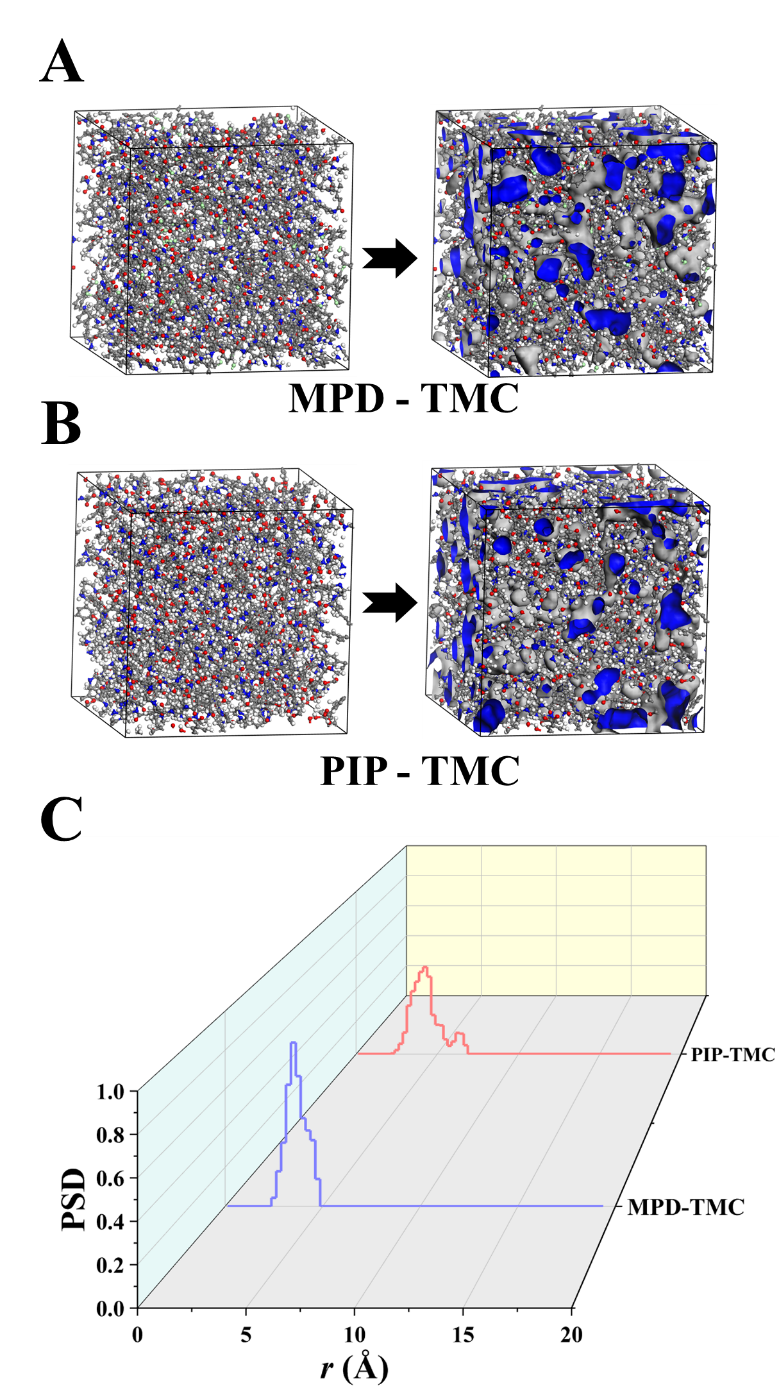


**Fig. S6. Conventional crosslinking procedures without explicit solvent molecules.** (A) MPD-TMC system. (B) PIP-TMC system. (C) Pore size distribution (PSD) of the two PA membranes obtained from (A) and (B).

We also performed a simplified crosslinking process without explicitly including the two solvents, as proposed previously (*33*). Following this method, the polyamide networks generated from the MPD-TMC and PIP-TMC systems showed similar crosslinking degrees and pore size distributions **(Fig. S6**), which could not explain the differences in the free volume/pore size of RO and NF membranes. Therefore, the aqueous interface with both solvents considered in our *in-situ* crosslinking method is critical for the differences between the RO and NF membranes.


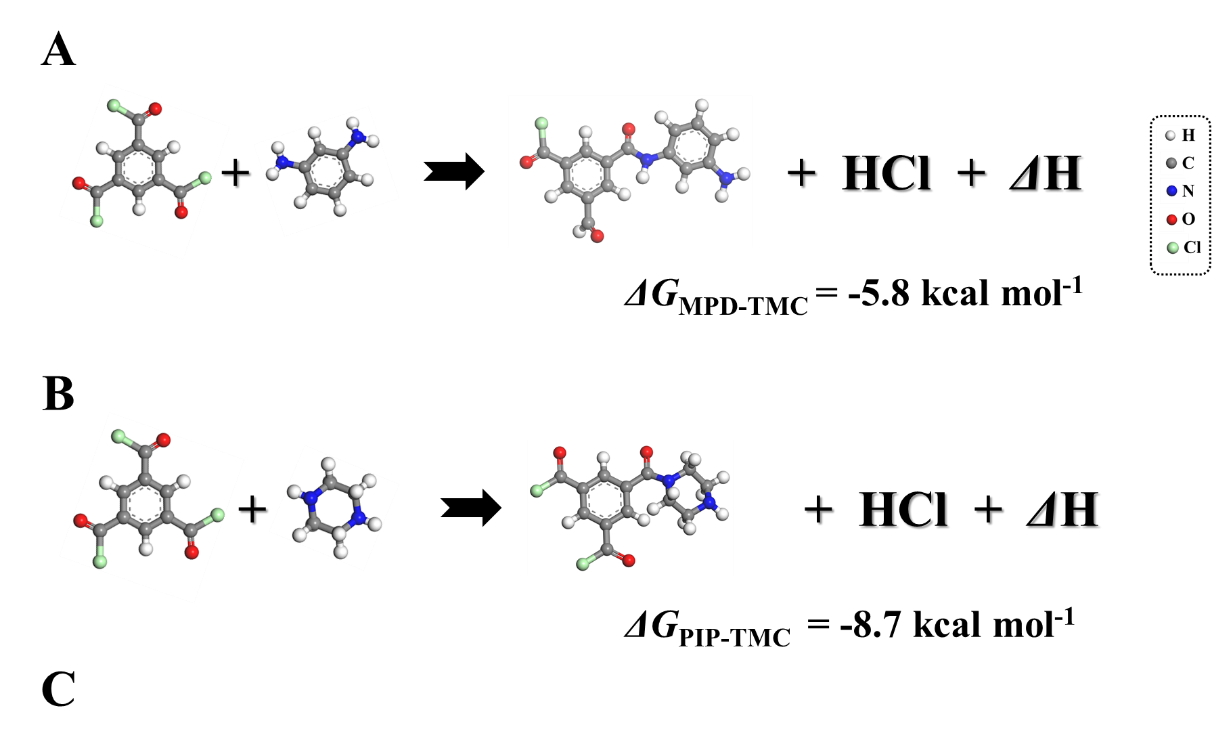

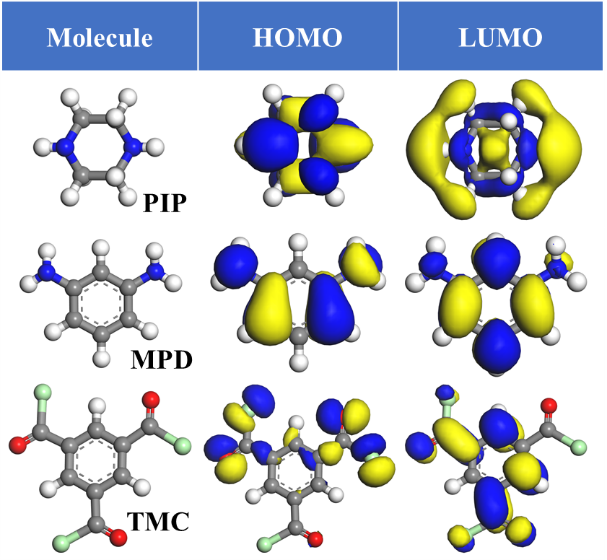


**Fig. S7. Reaction energy and reactivity of the amine monomer with TMC.** (A) Polymerization between MPD and TMC, and the reaction energy Δ*G*. (B) Polymerization between PIP and TMC, and the reaction energy Δ*G*. (C) The highest occupied molecular orbital (HOMO) and lowest unoccupied molecular orbital (LUMO) of different reactive monomers.

Combined with Table S4, the frontier orbital gap indicates a higher chemical reactivity of the PIP monomer with TMC, which is consistent with previous experimental measurements showing that the reactivity of PIP with TMC is 10^4^ – 10^5^ L mol^-1^ s^-1^, two orders of magnitude higher than that of MPD with TMC (110 L mol^-1^ s^-1^) (*25*).


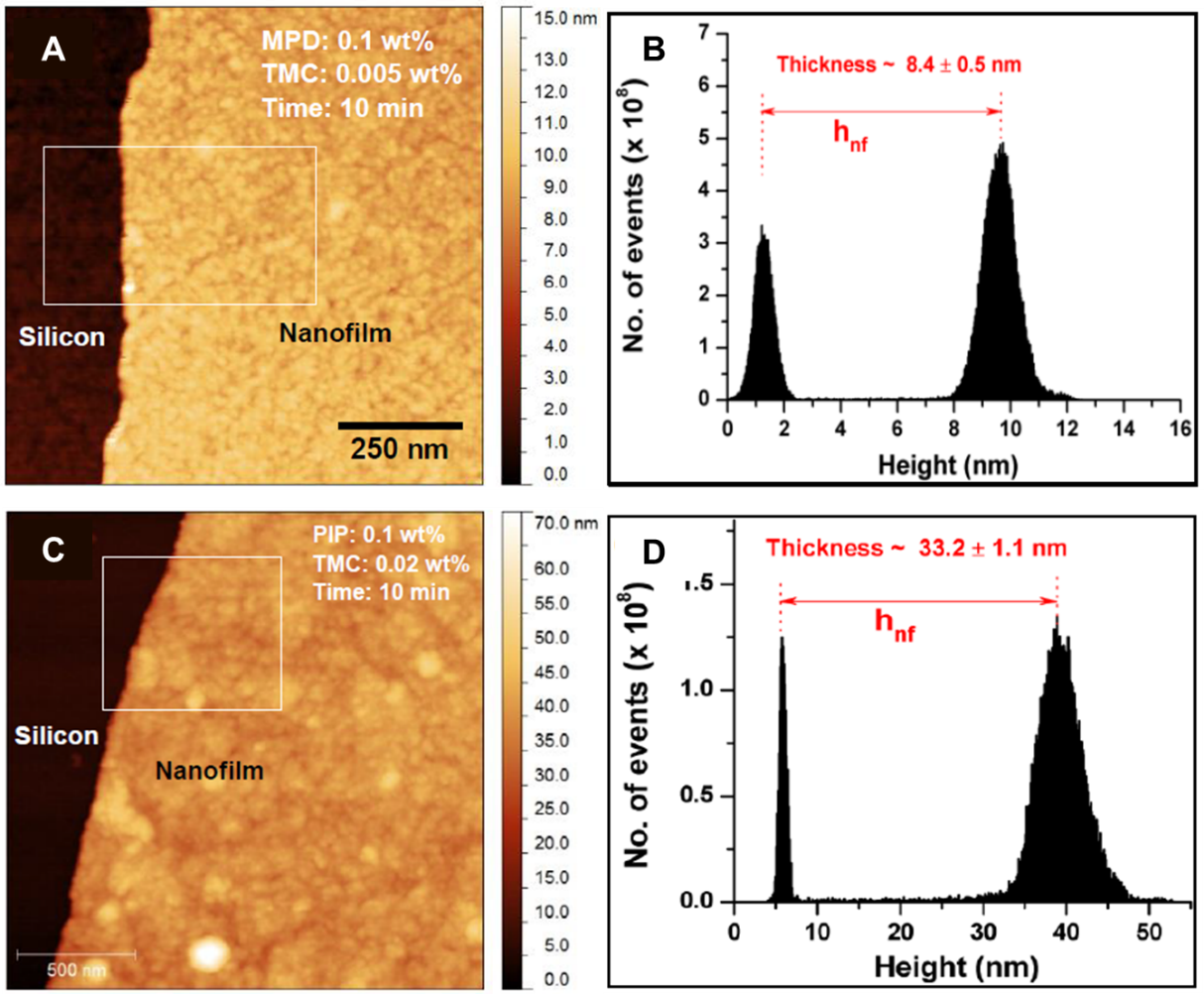


**Figure S8. Morphology and thickness measurement of free-standing polyamide nanofilms.^[20]^** AFM height images and height profiles (for marked regions in height images) of (A, B) MPD-TMC system; (C, D) PIP-TMC system. IP reaction time: 10 min.

The freestanding PA membranes are formed after the IP reactions for 10 mins, much longer than the time needed for the incipient film formation. Therefore, the thicknesses of these mature PA membranes fabricated in both MPD-TMC and PIP-TMC systems are larger than those of the incipient films, as illustrated in Fig. 2.


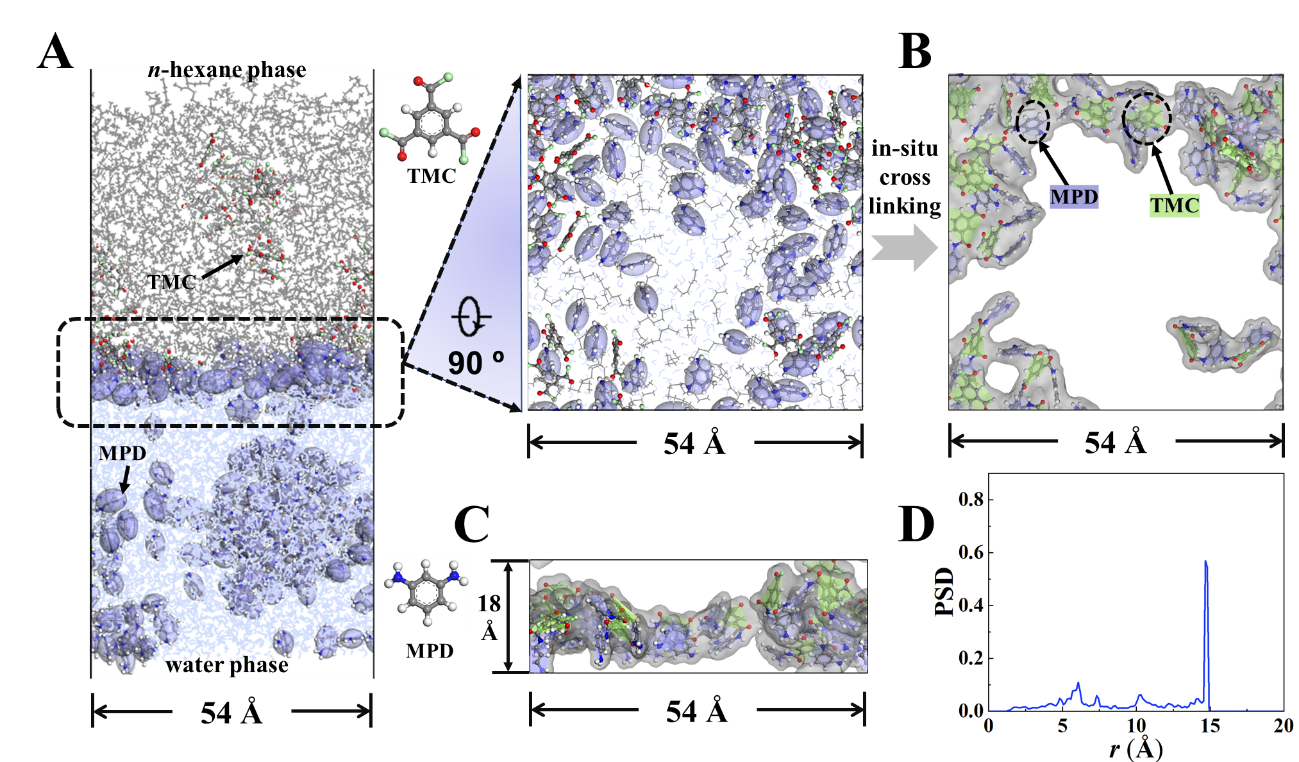


**Fig. S9. Incipient film formation in M3 system after 1 ns of monomer diffusion.** (A) Snapshot of the interfacial system after 1 ns of equilibrium diffusion (left), with the extracted reaction zone (right). The initial M3 system contains 200 MPD molecules in the water phase and 50 TMC molecules in the *n*-hexane phase. Other molecules that distribute outside the reaction zone are set as invisible on the right panel. (B) Snapshot of the PA film after *in-situ* crosslinking from (A). The MPD and TMC fragment in the PA network are marked in lavender and green, respectively. (C) Thickness of the incipient PA film obtained in (B). (D) PSD of the incipient PA film obtained in (B).


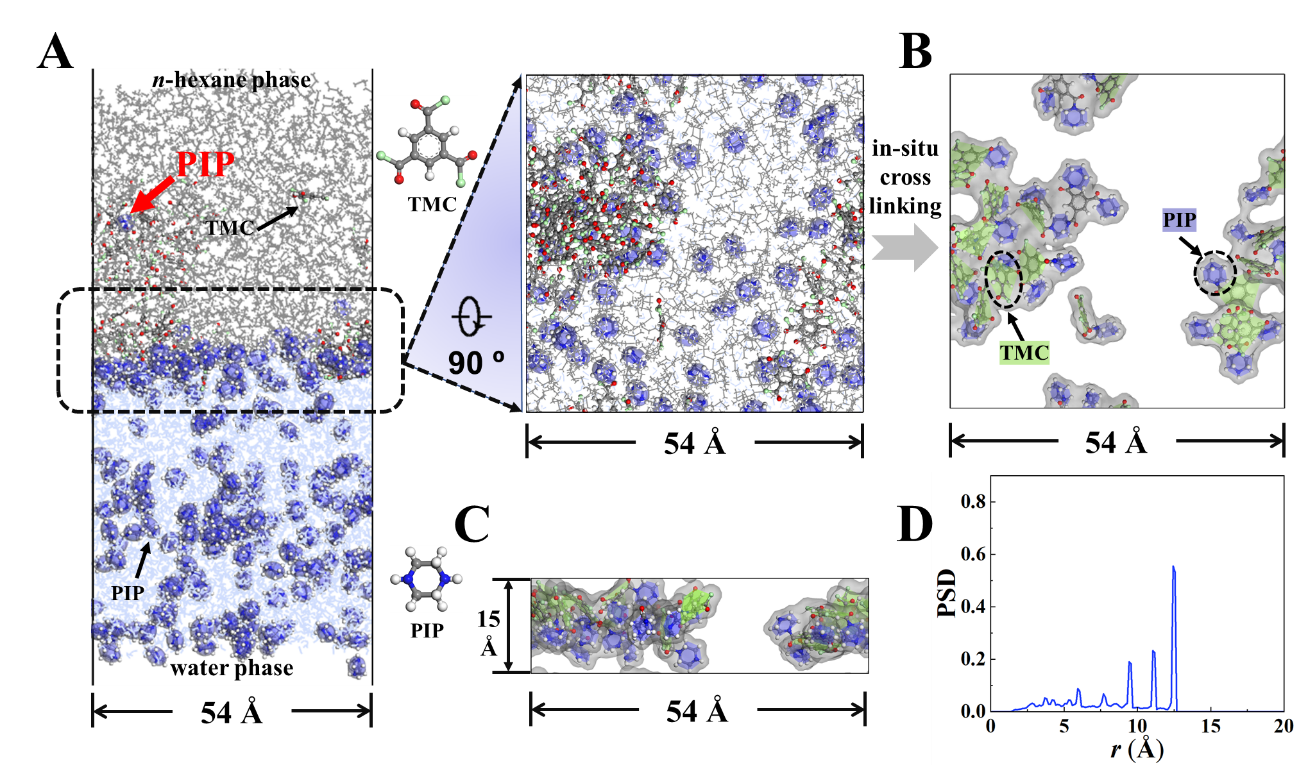


**Fig. S10. Incipient film formation in P3 system after 1 ns of monomer diffusion**. (A) Snapshot of the interfacial system after 1 ns of equilibrium diffusion (left), with the extracted reaction zone (right). The initial P3 system contains 200 PIP molecules in the water phase and 50 TMC molecules in the *n*-hexane phase. Other molecules distributed outside the reaction zone are set as invisible on the right panel. (B) Snapshot of the PA film after *in-situ* crosslinking from (A). The PIP and TMC fragment in the PA network are marked in blue and green, respectively. (C) Thickness of the incipient PA film obtained in (B). (D) PSD of the incipient PA film obtained in (B).


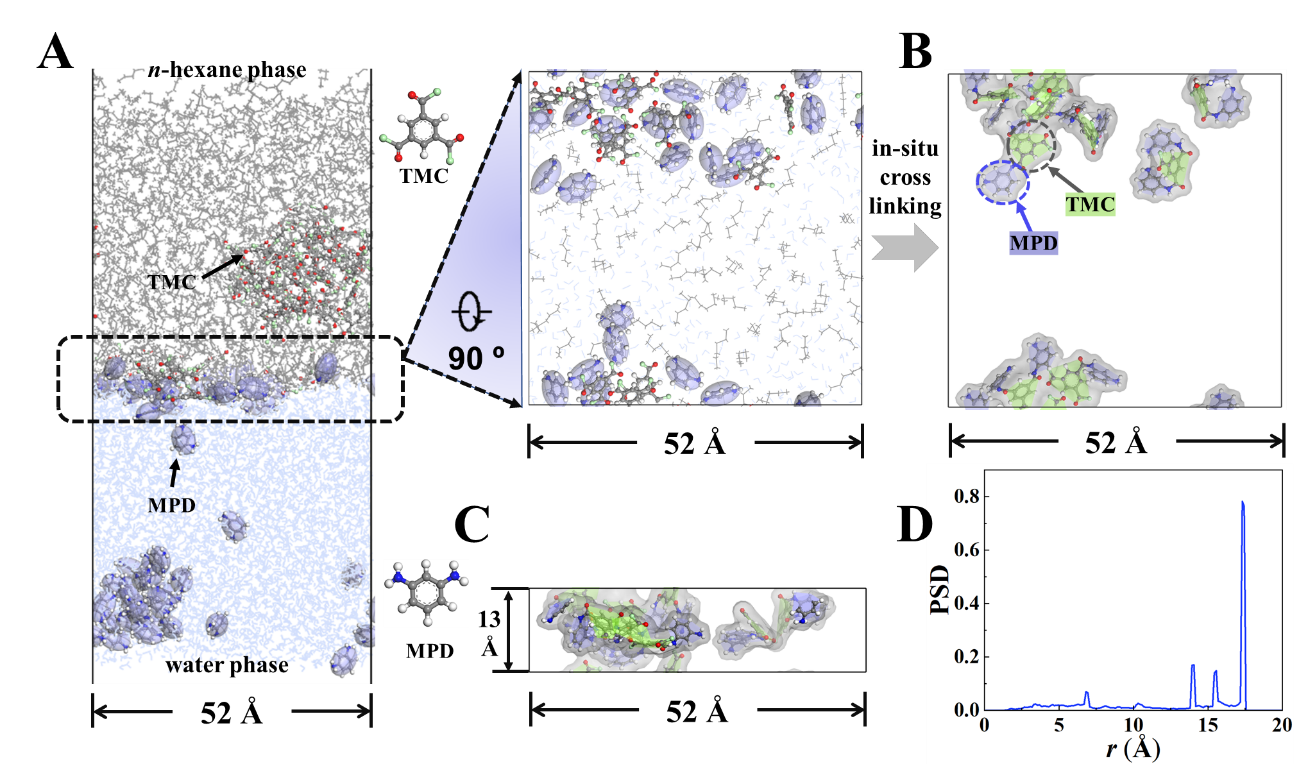


**Fig. S11. Incipient film formation in M1 system after 5 ns of monomer diffusion.** (A) Snapshot of the interfacial system after 5 ns of equilibrium diffusion (left), with the extracted reaction zone (right). The initial M1 system contains 50 MPD molecules in the water phase and 50 TMC molecules in the *n*-hexane phase. Other molecules distributed outside the reaction zone are set as invisible on the right panel. (B) Snapshot of the PA film after *in-situ* crosslinking from (A). The MPD and TMC fragment in the PA network are marked in lavender and green, respectively. (C) Thickness of the incipient PA film obtained in (B). (D) PSD of the incipient PA film obtained in (B).


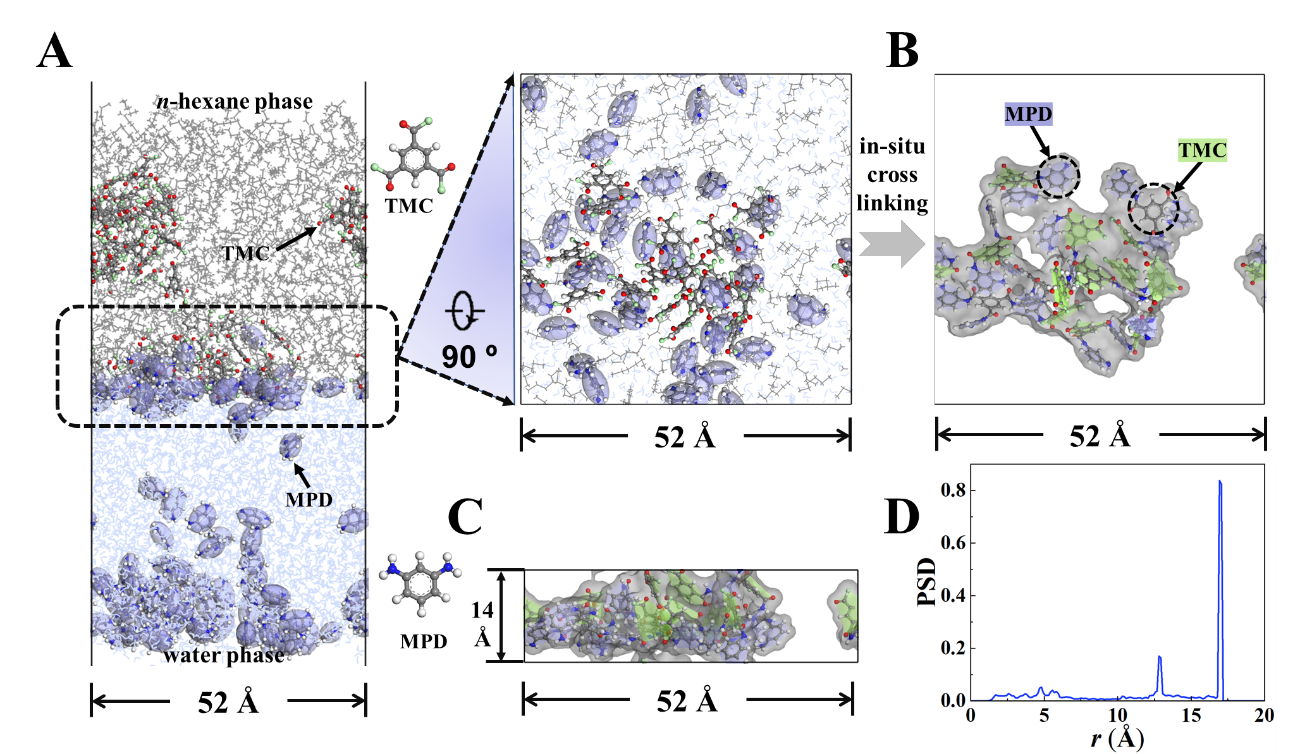


**Fig. S12. Incipient film formation in M2 system after 5 ns of monomer diffusion.** (A) Snapshot of the interfacial system after 5 ns of equilibrium diffusion (left), with the extracted reaction zone (right). The initial M2 system contains 100 MPD molecules in the water phase and 50 TMC molecules in the *n*-hexane phase. Other molecules distributed outside the reaction zone are set as invisible on the right panel. (B) Snapshot of the PA film after *in-situ* crosslinking from (A). The MPD and TMC fragment in the PA network are marked in lavender and green, respectively. (C) Thickness of the incipient PA film obtained in (B). (D) PSD of the incipient PA film obtained in (B).

**
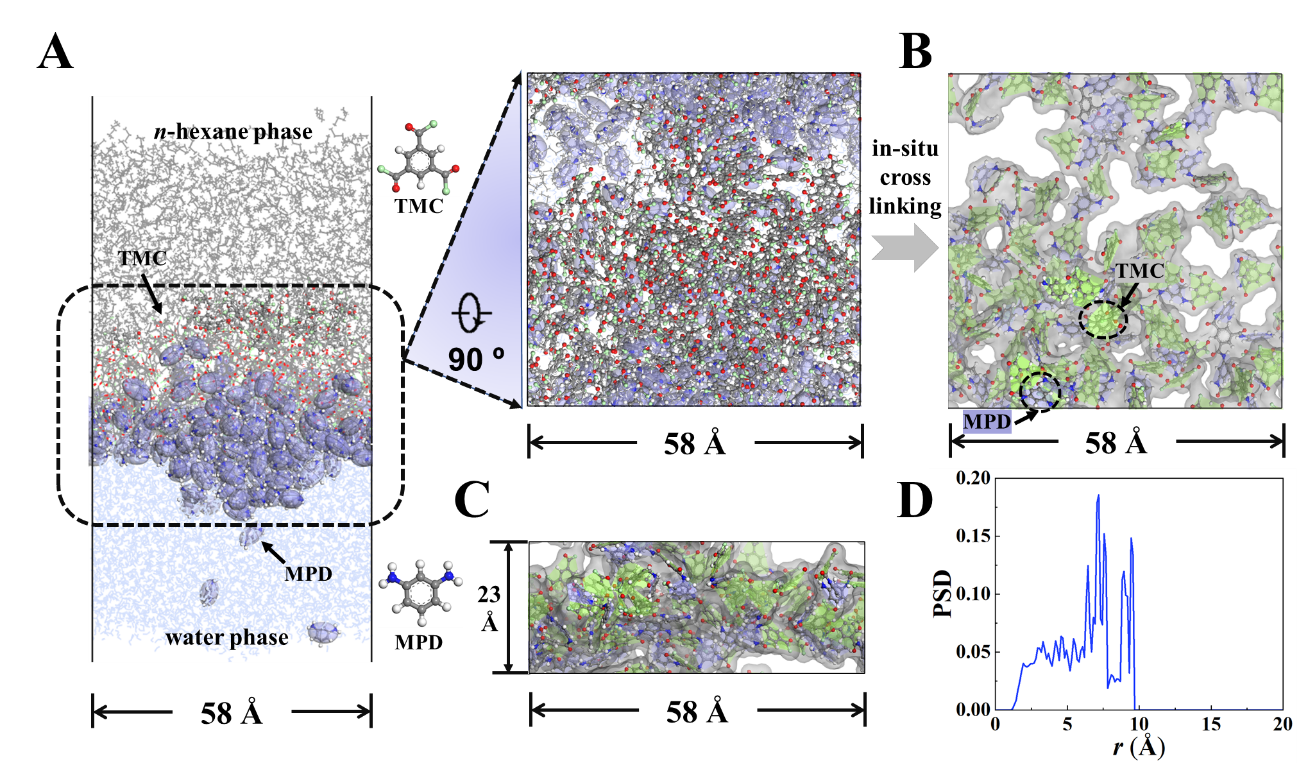
**

**Fig. S13. Incipient film formation in M4 system after 5 ns of monomer diffusion.** (A) Snapshot of the interfacial system after 5 ns of equilibrium diffusion (left), with the extracted reaction zone (right). The initial M4 system contains 200 MPD molecules in the water phase and 200 TMC molecules in the *n*-hexane phase. Other molecules distributed outside the reaction zone are set as invisible on the right panel. (B) Snapshot of the PA film after *in-situ* crosslinking from (A). The MPD and TMC fragment in the PA network are marked in lavender and green, respectively. (C) Thickness of the incipient PA film obtained in (B). (D) PSD of the incipient PA film obtained in (B).


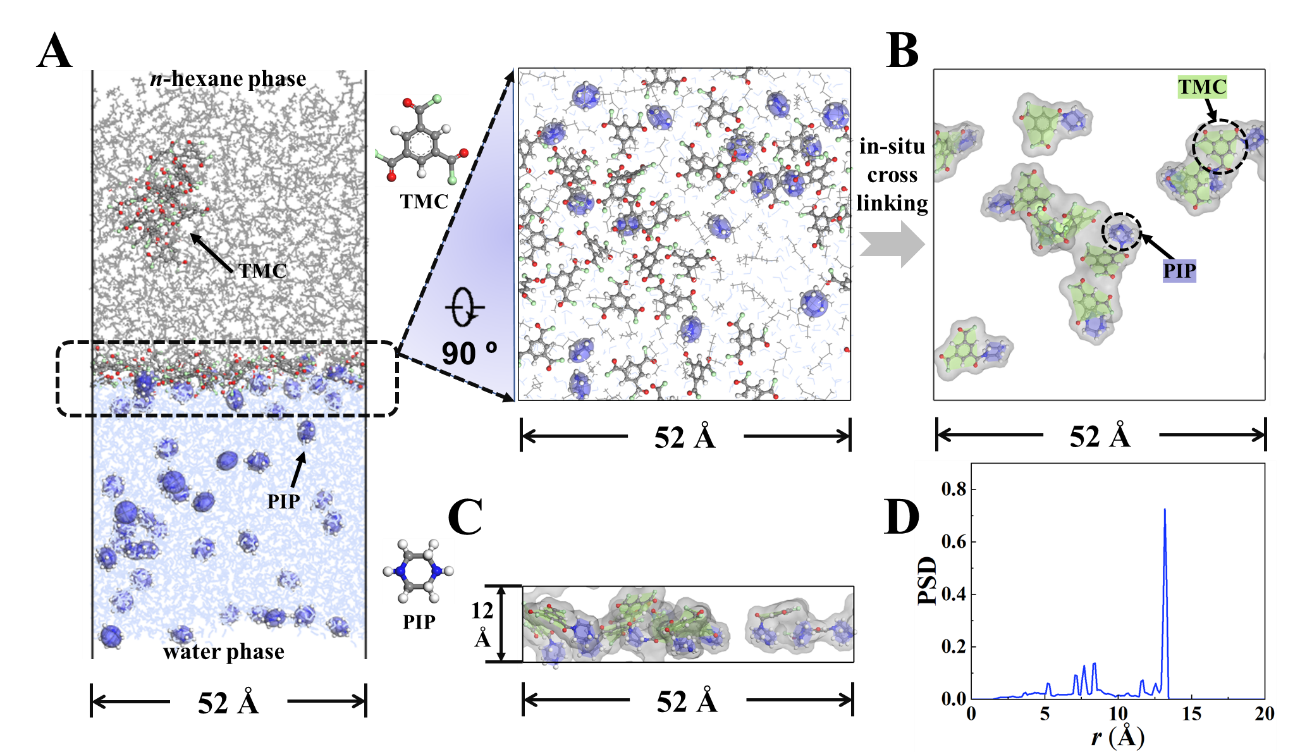


**Fig. S14. Incipient film formation in P1 system after 5 ns of monomer diffusion**. (A) Snapshot of the interfacial system after 5 ns of equilibrium diffusion (left), with the extracted reaction zone (right). The initial P1 system contains 50 PIP molecules in the water phase and 50 TMC molecules in the *n*-hexane phase. Other molecules distributed outside the reaction zone are set as invisible on the right panel. (B) Snapshot of the PA film after *in-situ* crosslinking from (A). The PIP and TMC fragment in the PA network are marked in blue and green, respectively. (C) Thickness of the incipient PA film obtained in (B). (D) PSD of the incipient PA film obtained in (B).


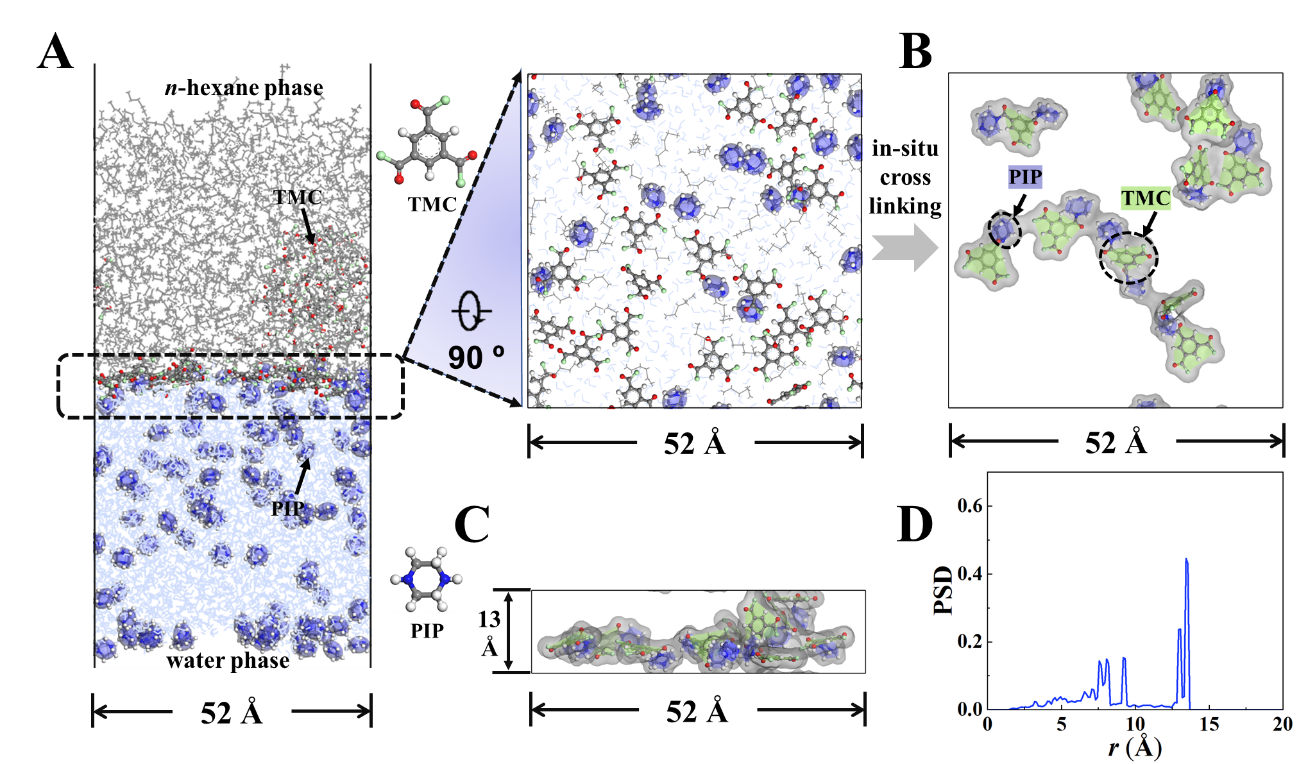


**Fig. S15. Incipient film formation in P2 system after 5 ns of monomer diffusion**. (A) Snapshot of the interfacial system after 5 ns of equilibrium diffusion (left), with the extracted reaction zone (right). The initial P2 system contains 100 PIP molecules in the water phase and 50 TMC molecules in the *n*-hexane phase. Other molecules distributed outside the reaction zone are set as invisible on the right panel. (B) Snapshot of the PA film after *in-situ* crosslinking from (A). The PIP and TMC fragment in the PA network are marked in blue and green, respectively. (C) Thickness of the incipient PA film obtained in (B). (D) PSD of the incipient PA film obtained in (B).


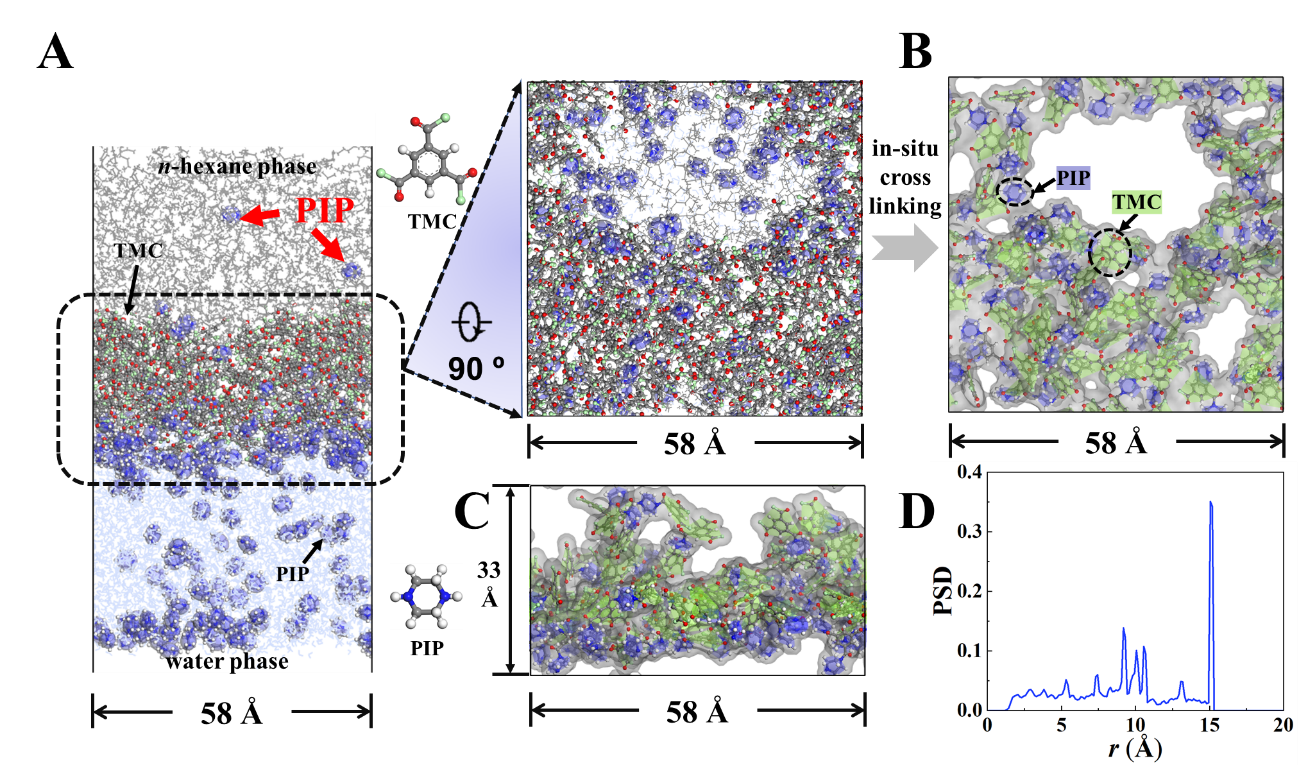


**Fig. S16. Incipient film formation in P4 system after 5 ns of monomer diffusion**. (A) Snapshot of the interfacial system after 5 ns of equilibrium diffusion (left), with the extracted reaction zone (right). The initial P4 system contains 200 PIP molecules in the water phase and 200 TMC molecules in the *n*-hexane phase. Other molecules distributed outside the reaction zone are set as invisible on the right panel. (B) Snapshot of the PA film after *in-situ* crosslinking from (A). The PIP and TMC fragment in the PA network are marked in blue and green, respectively. (C) Thickness of the incipient PA film obtained in (B). (D) PSD of the incipient PA film obtained in (B).


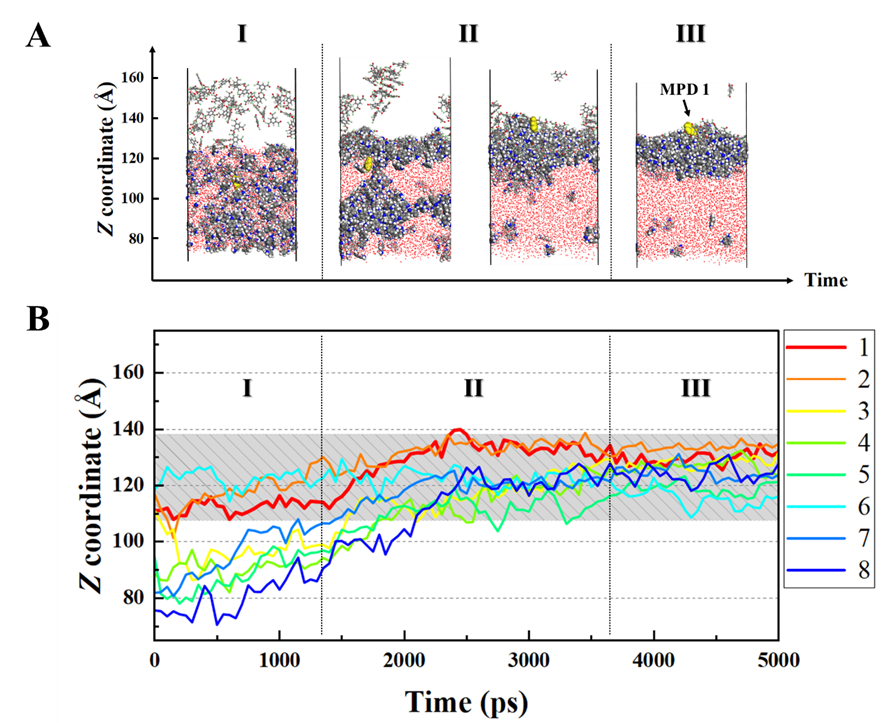


**Fig. S17. Trans-interface migration of MPD molecules.** (A) Snapshots of a selected MPD molecule (in yellow) in different sub-processes (I, II and III) during the whole diffusion course from the water phase to the organic side of the interface. Water phase is represented in red on the bottom and *n*-hexane phase is on the top (*n*-hexane molecules are set invisible). The diffusion coefficients of this MPD molecule in different sub-processes are listed in Table S5. (B) The evolution of *Z* coordinates of 8 selected MPD molecules (in different colors) during their diffusion courses. The red curve corresponds to the dynamic movement (perpendicular to the interface) of MPD 1 in (A). The equilibrium interfacial region is represented in grey shadow.


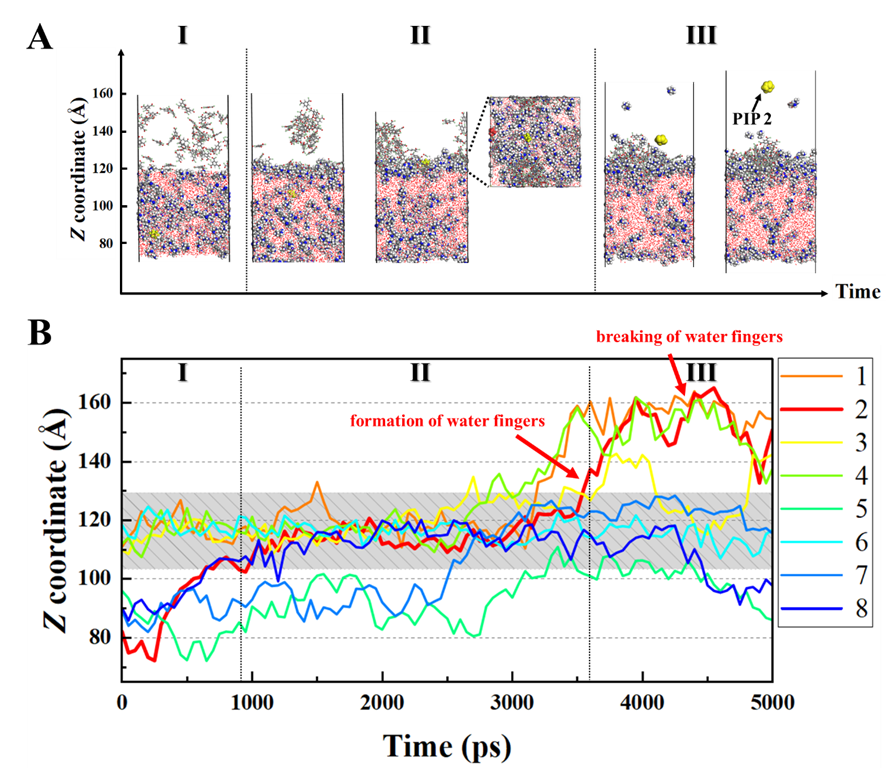


**Fig. S18. Trans-interface migration of PIP molecules.** (A) Snapshots of a selected PIP molecule (in yellow) in different sub-processes (I, II and III) during the whole diffusion course from the water phase to the organic side of the interface. Water phase is represented in red on the bottom and *n*-hexane phase is on the top (*n*-hexane molecules are set invisible). The diffusion coefficients of this PIP molecule in different sub-processes are listed in Table S6. (B) The evolution of *Z* coordinates of 8 selected PIP molecules (in different colors) during their diffusion courses. The red curve corresponds to the dynamic movement (perpendicular to the interface) of PIP 2 in (A). The equilibrium interfacial region is represented in grey shadow. The formation and breaking of a water finger are indicated in sub-processes II and III.


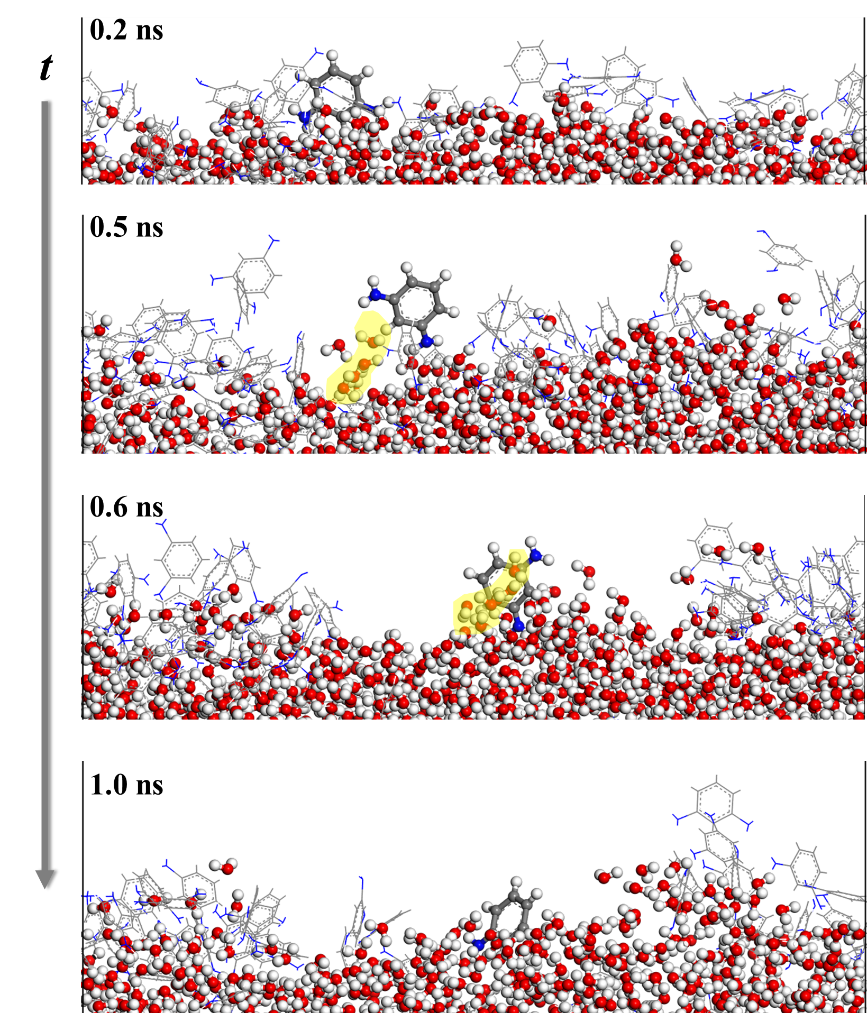


**Fig. S19. Snapshots of dynamic interactions between a targeted MPD molecule and transient water fingers (highlighted in yellow) in M3 system.**


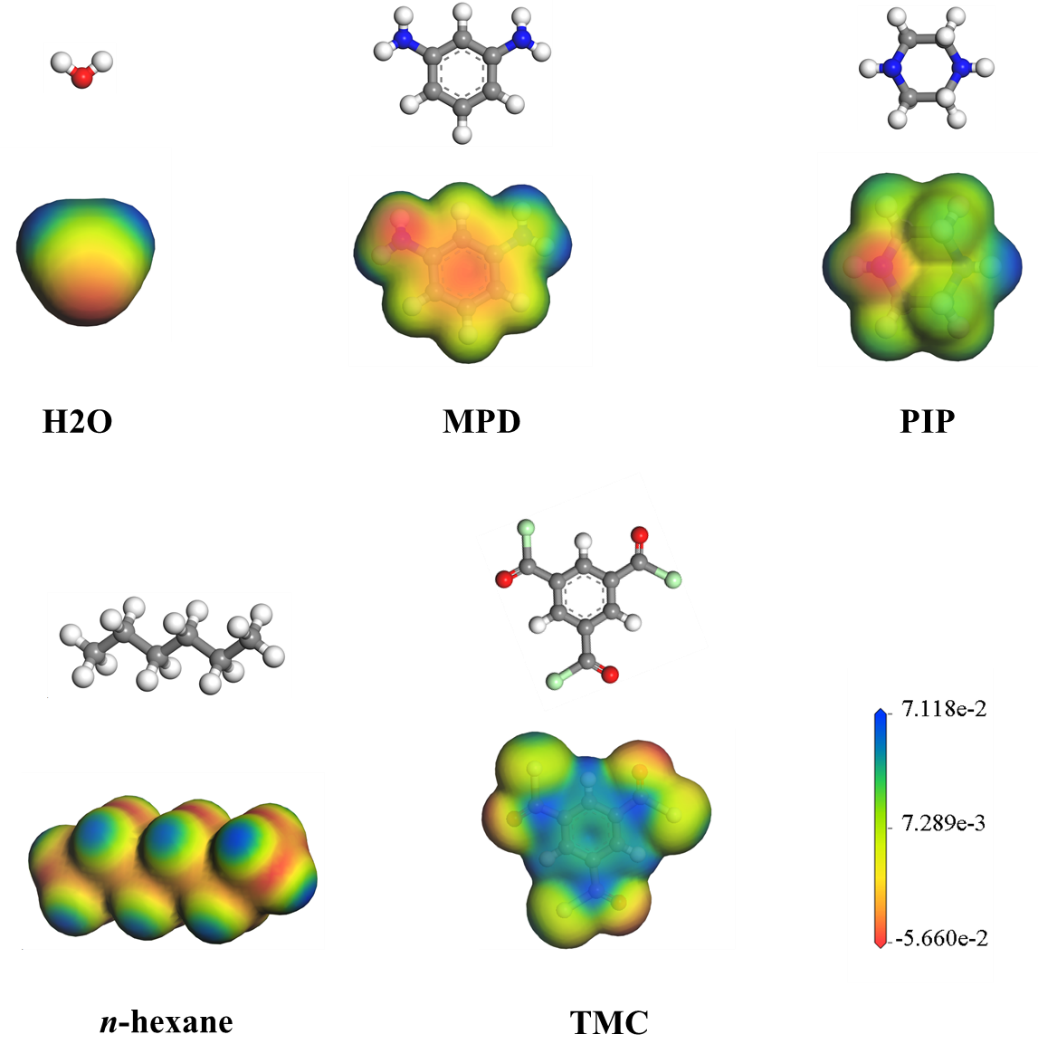


**Fig. S20. Electrostatic potentials (ESP) of different molecules in the interfacial polymerization system.**

**
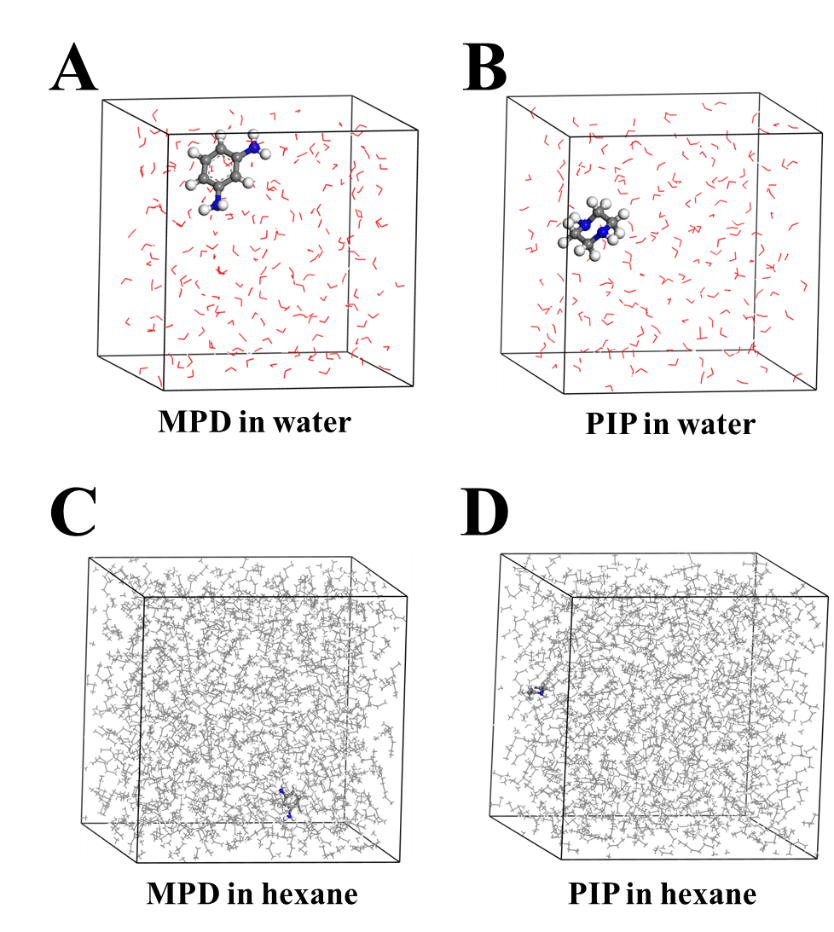
**

**Fig. S21. MD models of amine monomers in different solvent solutions.** (A) One MPD molecule in pure water containing 200 water molecules. (B) One PIP molecule in pure water containing 200 water molecules. (C) One MPD molecule in *n*-hexane solution containing 500 *n*-hexane molecules. (D) One PIP molecule in *n*-hexane solution containing 500 *n*-hexane molecules. The corresponding diffusion coefficients and binding energies are listed in Table S7 and Table S8, respectively.


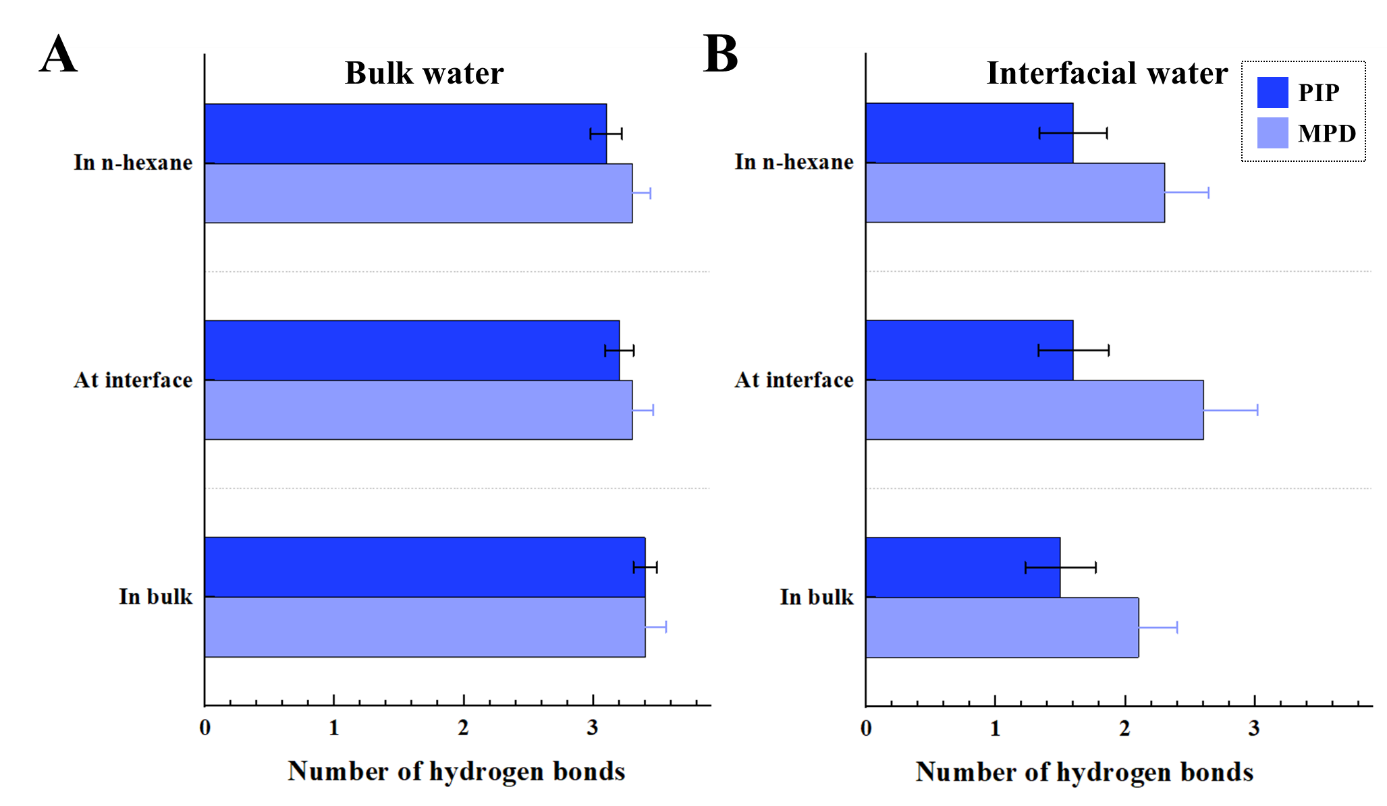


**Fig. S22. Number of hydrogen bonds for water molecules.** (A) Hydrogen bond numbers of bulk water molecules when the amine monomer (MPD in lavender and PIP in blue) is in different positions. (B) Hydrogen bond numbers of interfacial water molecules when the amine monomer (MPD in lavender and PIP in blue) is in different positions. The *Y* axes indicate different positions of amine monomers.

The calculated average number of hydrogen bonds formed among bulk water molecules is 3.4 per molecule in MPD system and 3.3 per molecule in PIP system, respectively. Both values are close to the literature value of hydrogen bonded water molecules in the liquid water (*14*, *15*), indicating a stable hydrogen bonding network of liquid water in spite of the positions of the amine monomers. In contrast, water molecules at the interface have to sacrifice, on average, around 1 hydrogen bond per water molecule in MPD system and 1.6 hydrogen bond per water molecule in PIP system. Specifically, the average number of hydrogen bonds of interfacial water molecules is larger in the MPD system than that in the PIP system. When the amine monomer is in the bulk phase or in the *n*-hexane phase, the average number of hydrogen bonds formed among interfacial water molecules is 2.1 per molecule in MPD system and 1.5 per molecule in PIP system. This value increases to 2.6 in MPD system and 1.6 in PIP system when the amine monomer transfers to the interface, indicating a stronger hydrogen binding network between MPD and water molecules.


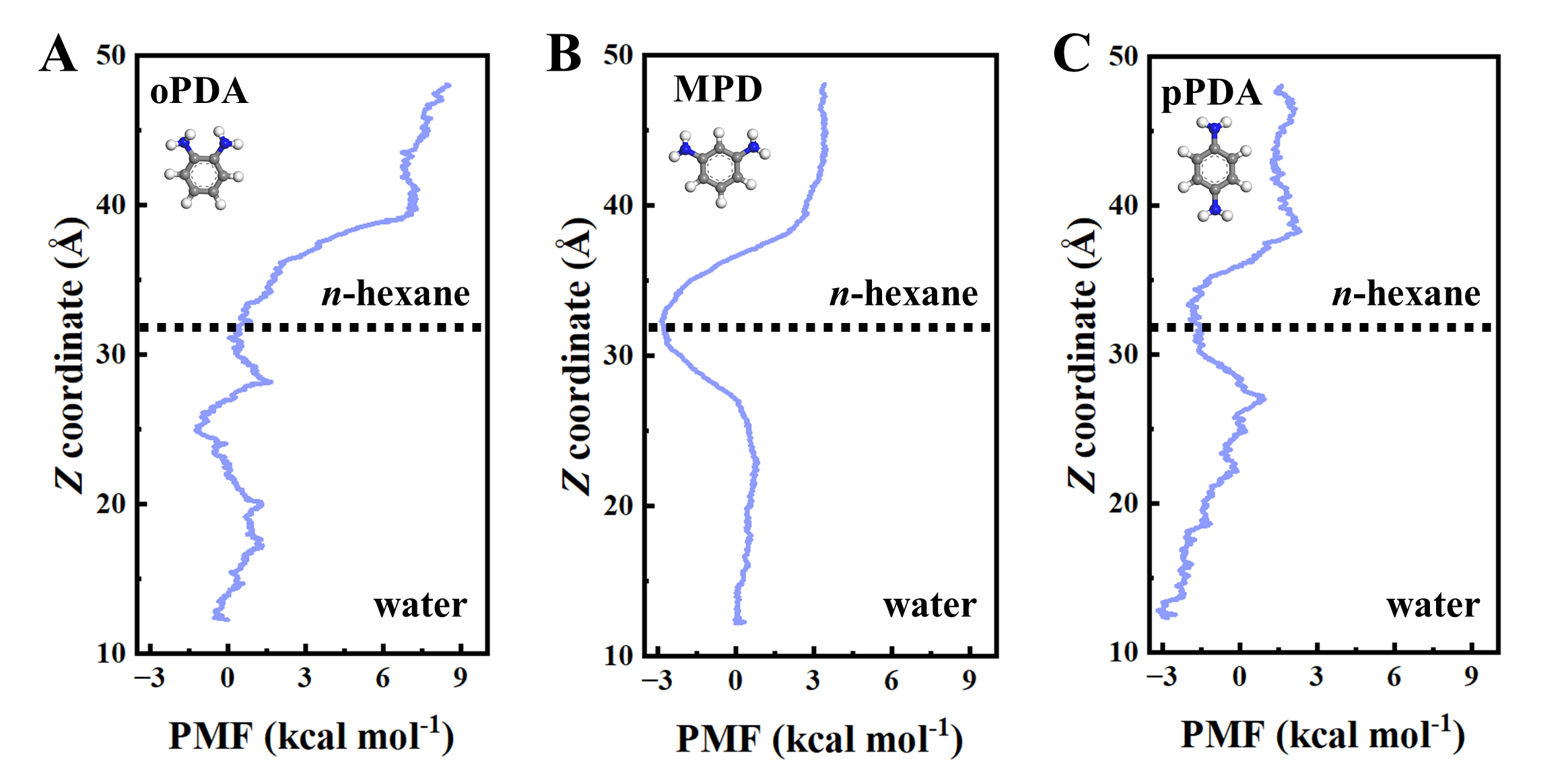


**Figure S23. PMF profiles for aromatic diamines oPDA (A), MPD (B), and pPDA (C) along the *Z*-coordinate across the water/*n*-hexane interface.** Dashed lines indicate the aqueous-organic interfacial boundaries.

We conducted MD simulations on the two monomers and found that, the linear structure of p-PDA favors different interfacial orientations and hydrogen bonding configurations compared to MPD, while o-PDA, with adjacent amine groups, forms intramolecular hydrogen bonds that alter its hydration shell and interfacial dynamics. These structural variations are likely to influence both the energy barriers observed in PMF profiles and the nature of water finger interactions (Figure S23). Investigating such isomeric effects could enrich our understanding of monomer-specific transport mechanisms and inform the rational design of functional monomers for IP.

**
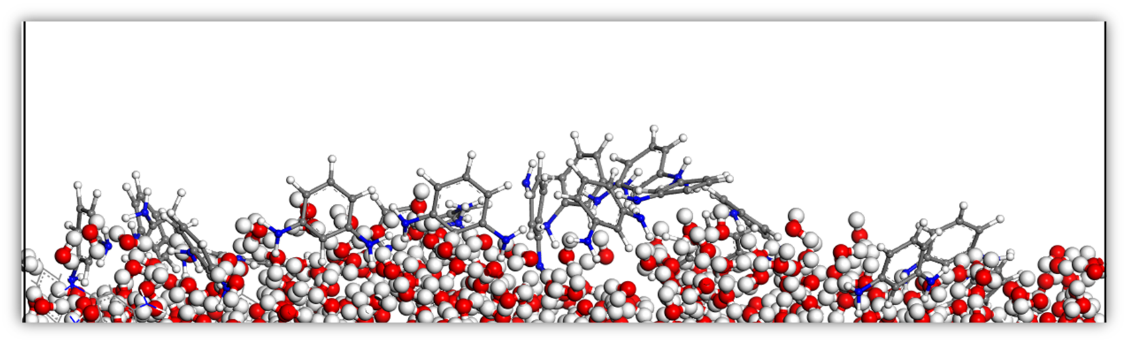
**

**Fig. S24. Snapshot of interfacial water and MPD molecules in M1 system during 5 ns of equilibrium diffusion.**

There were no apparent water fingers in this system because the concentration of MPD was very low. Most MPD molecules stayed in the solution and at the interface. The M1 system contained 50 MPD molecules in water and 50 TMC molecules in *n*-hexane.


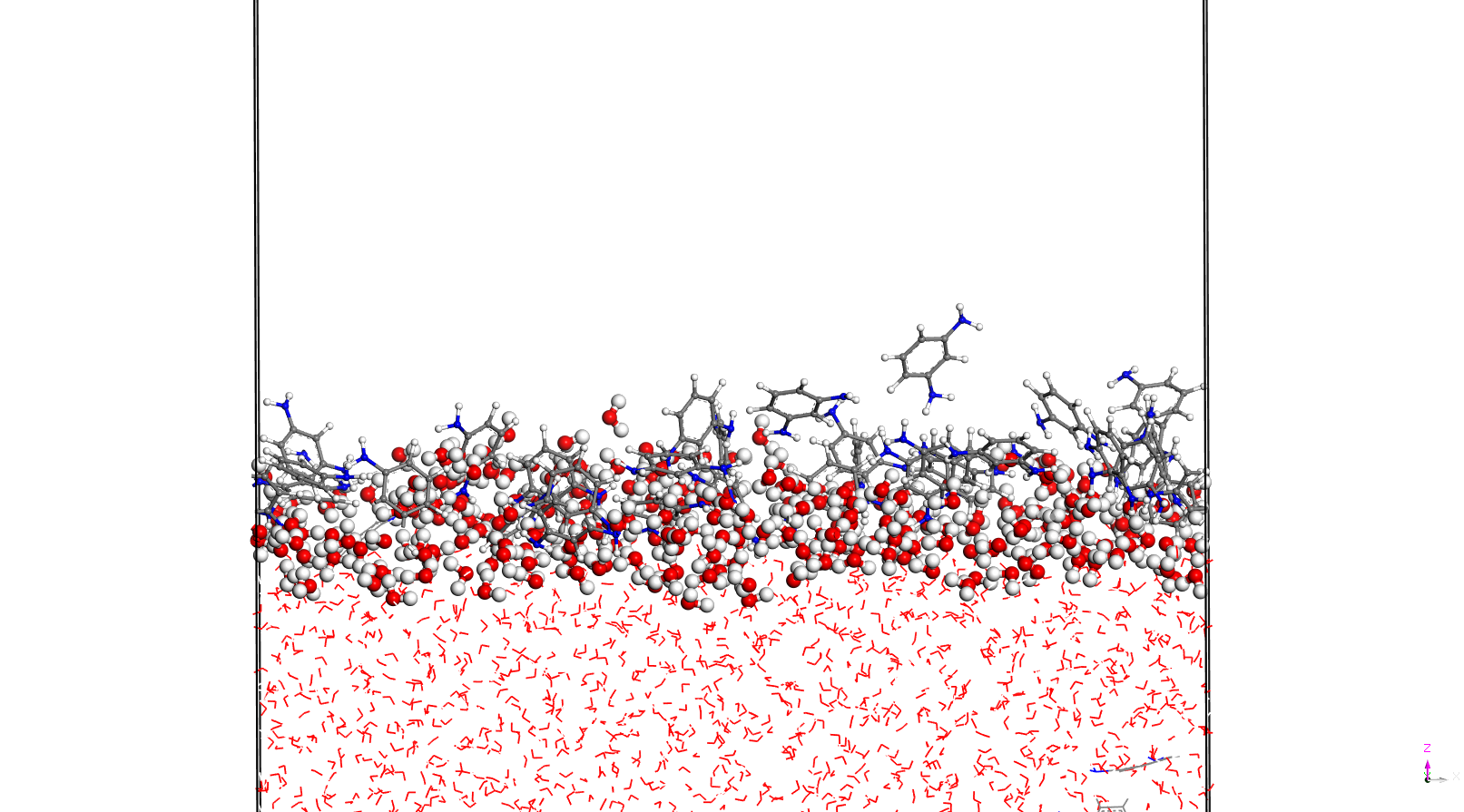


**Fig. S25. Snapshot of interfacial water and MPD molecules in M2 system during 5 ns of equilibrium diffusion.**

There were no apparent water fingers in this system because the concentration of MPD was very low. The M2 system contained 100 MPD molecules in water and 50 TMC molecules in *n*-hexane. However, some water molecules jump up and down frequently within a short distance from the interface over the entire course.


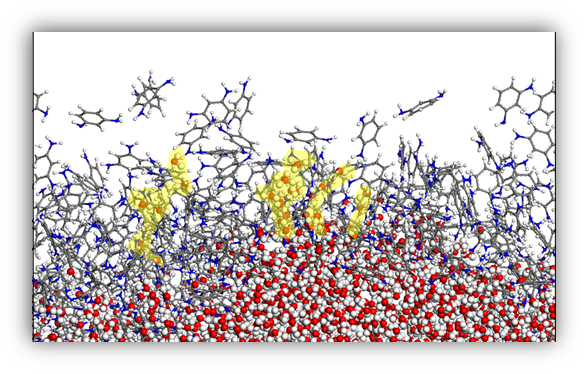


**Fig. S26. Snapshot of interfacial water and MPD molecules in M4 system during 5 ns of equilibrium diffusion. Water fingers are highlighted in yellow.**

The M4 system contained 200 MPD molecules in water and 200 TMC molecules in *n*-hexane. There were noticeable water fingers in this system, and the fraction of MPD molecules penetrating the *n*-hexane phase was relatively high compared to other MPD-TMC systems (M1 to M3).


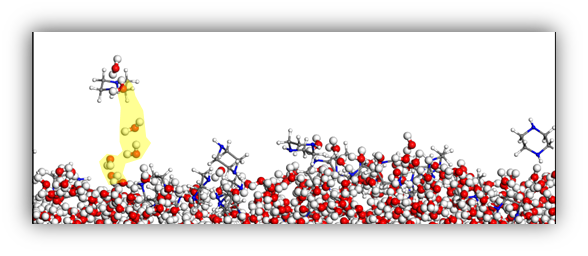


**Fig. S27. Snapshot of interfacial water and PIP molecules in P1 system during 5 ns of equilibrium diffusion. Water finger is highlighted in yellow.**

The P1 system contained 50 PIP and 50 TMC molecules in water and *n*-hexane. There were evident water fingers in this system, although the concentration of PIP was very low.


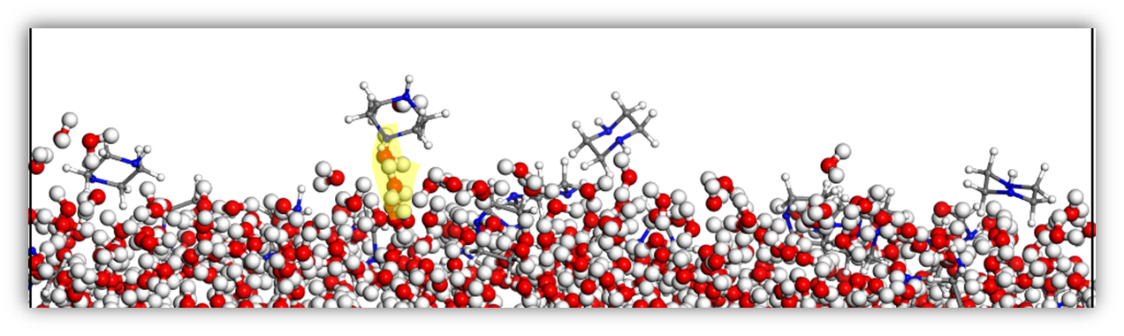


**Fig. S28. Snapshot of interfacial water and PIP molecules in P2 system during 5 ns of equilibrium diffusion. Water finger is highlighted in yellow.**

The P2 system contained 100 PIP molecules in water and 50 TMC molecules in *n*-hexane. There were clear water fingers in this system.


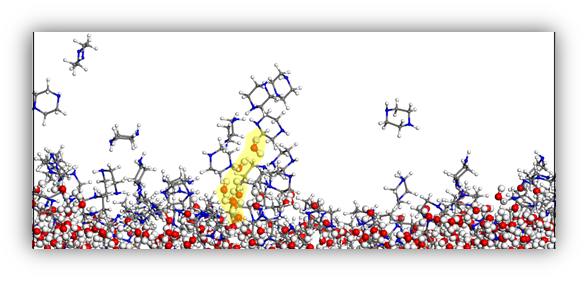


**Fig. S29. Snapshot of interfacial water and PIP molecules in P4 system during 5 ns of equilibrium diffusion. Water finger is highlighted in yellow.**

The P4 system contained 200 PIP molecules in water and 200 TMC molecules in *n*-hexane. There were noticeable water fingers in this system, and the fraction of PIP molecules penetrating the *n*-hexane phase was relatively high compared to other PIP-TMC systems (P1 to P3).


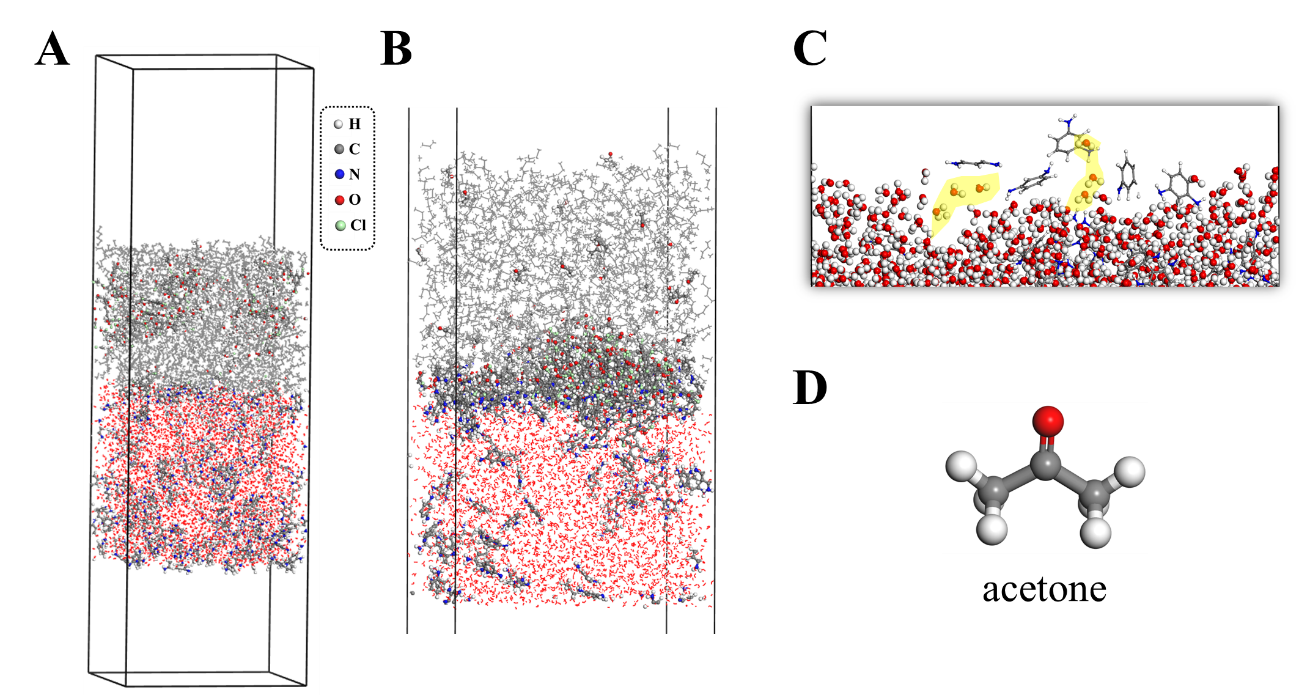


**Fig. S30. Snapshot of interfacial water and MPD molecules in co-solvent M2 system.** (A) Initial system. 100 molecules of acetone were added to the *n*-hexane phase as a co-solvent. (B) After 5 ns of equilibrium diffusion. (C) Partial enlargement of the interfacial zone. Only water and MPD molecules are shown, and other molecules are set invisible. Water fingers are highlighted in yellow. (D) Molecular structure of the co-solvent acetone. For comparison, M2 system without co-solvent has no apparent water fingers as shown in Fig. S25.

Interfacial tension also plays a critical role in modulating water finger behavior. The introduction of interfacial tension modifiers—such as co-solvents (Figure S30) or micellar surfactant solutions (Figure S31)—significantly amplifies the finger effect. These additives lower interfacial tension, allowing greater amplitude of interfacial undulations. This strategy aligns with the broader concept that water can reorganize its hydrogen-bonding network to induce interfacial instability.


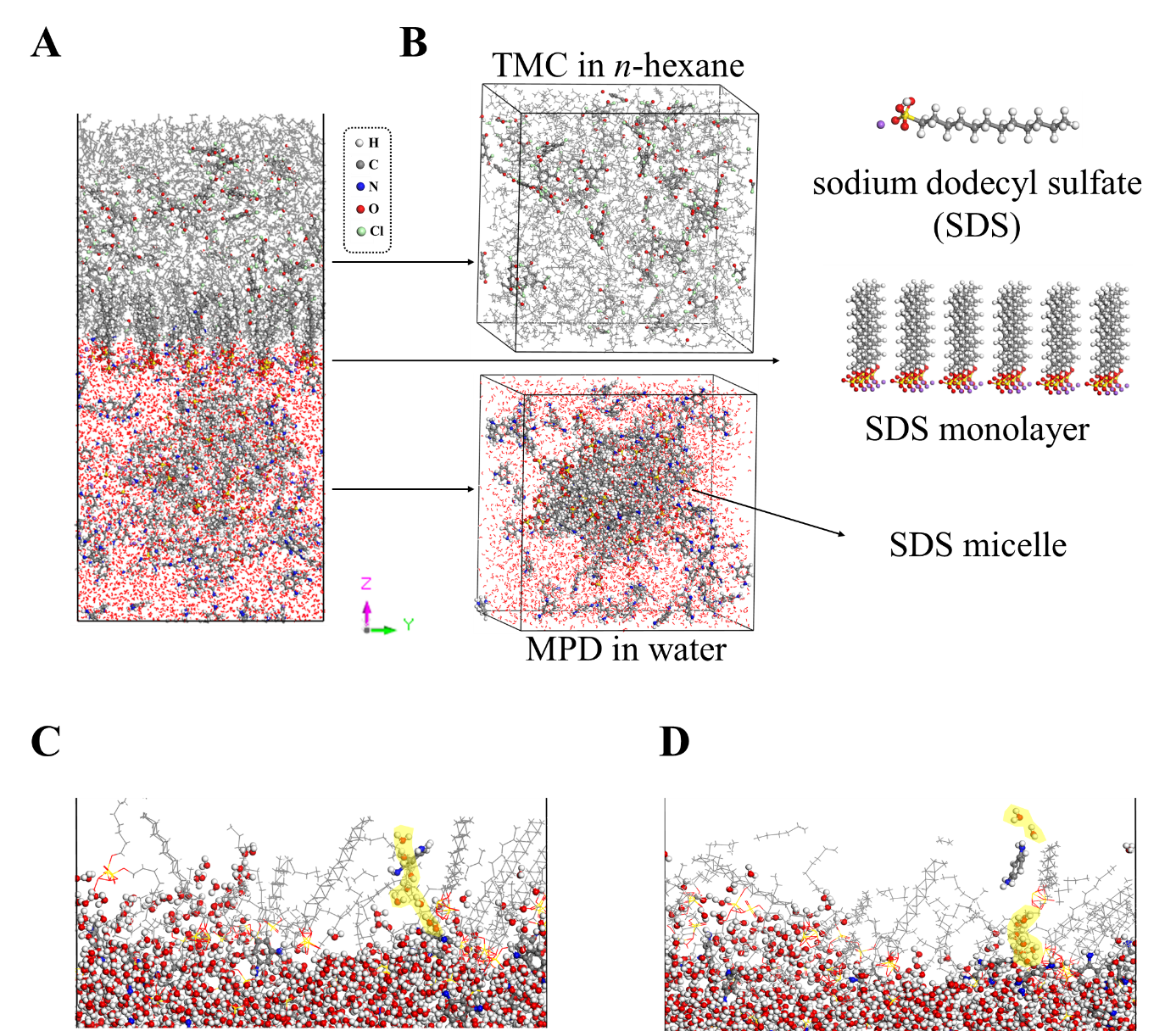


**Fig. S31. Snapshot of interfacial water and MPD molecules in micellar M2 system.** (A) Initial system. Solvent molecules (water and *n*-hexane) are shown in lines. MPD, TMC and SDS molecules are shown in ball-and-stick models. (B) Composition of the micellar M2 system. A surfactant monolayer composed of 36 molecules of sodium dodecyl sulfate (SDS) added at the interface and an SDS micelle containing 50 SDS molecules with a radius of 15 Å was added underneath the SDS monolayer with a distance of 25 Å in M2 system. (C and D) Partial enlargement of the interfacial zone after 5 ns of equilibrium diffusion, evidencing the formation of a water finger (C) and the breaking of this water finger (D). Only water (in ball-and-stick model), MPD (in ball-and-stick model) and SDS (in linear model) molecules are shown, and other molecules are set invisible. Water fingers are highlighted in yellow. For comparison, M2 system without SDS monolayer and micelle has no apparent water fingers as shown in Fig. S25.


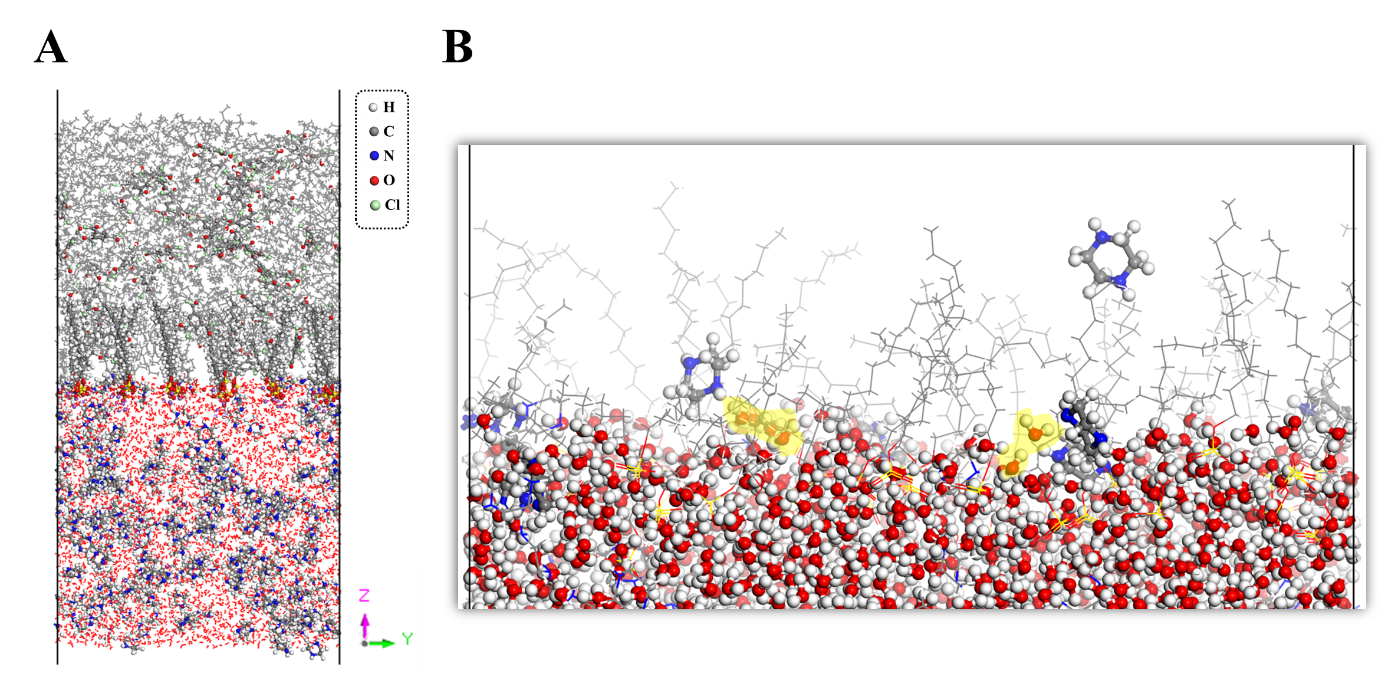


**Fig. S32. Snapshot of interfacial water and PIP molecules in monolayer P3 system.** (A) Initial system. A surfactant monolayer composed of 36 SDS molecules were added at the interface of P3 system. Solvent (water and *n*-hexane) molecules are shown in lines. PIP, TMC and SDS molecules are shown in ball-and-stick models. (B) Partial enlargement of the interfacial zone after 5 ns of equilibrium diffusion. Only water (in ball-and-stick model), PIP (in ball-and-stick model) and SDS (in linear model) molecules are shown, and other molecules are set invisible. Water fingers are highlighted in yellow. For comparison, P3 system without surfactant monolayer has apparent water fingers as shown in Fig. 3.


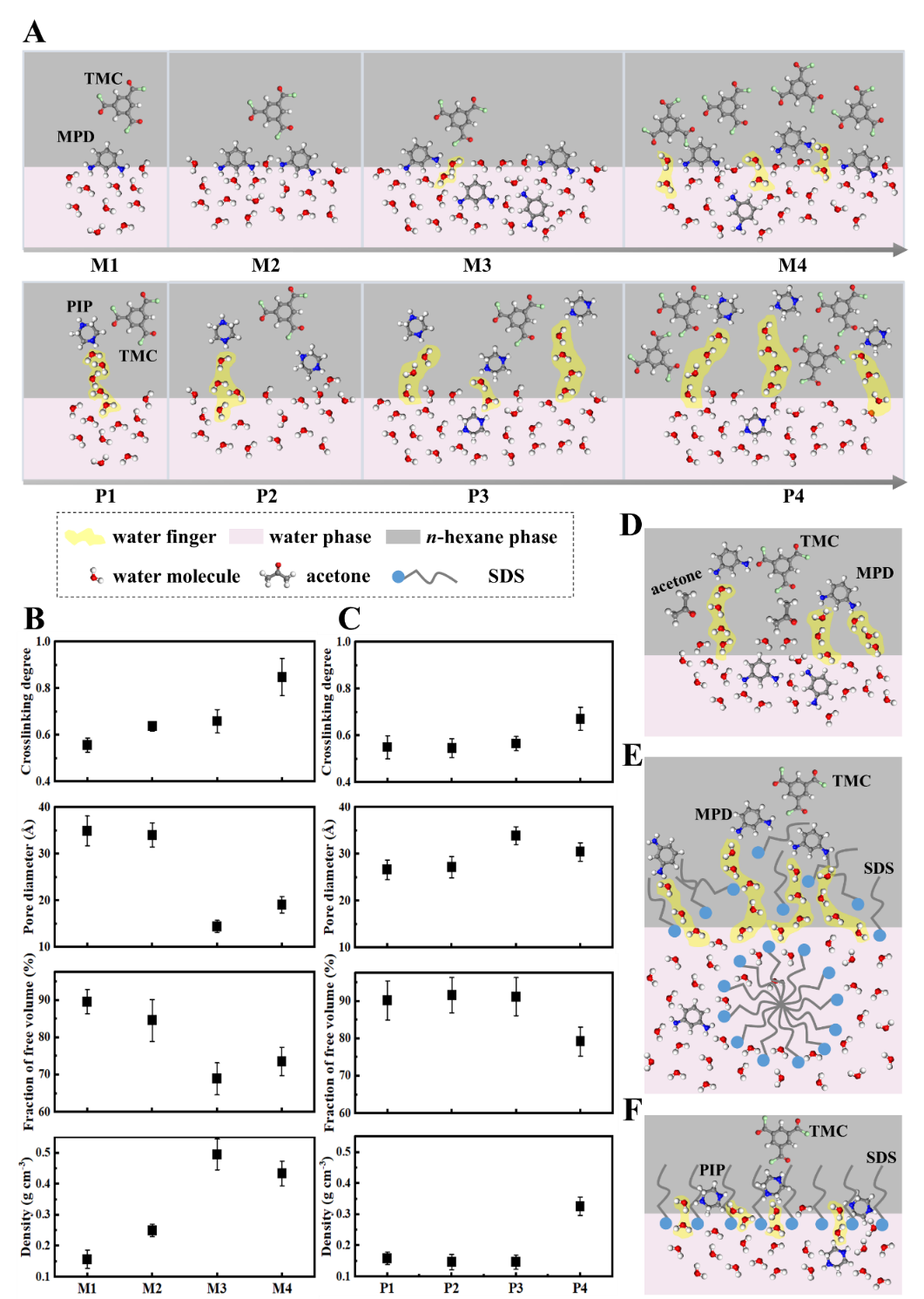


**Fig. S33. Role of water fingers in various systems.** (**A**) Schematic representation of water-amine monomer interactions at the water/*n*-hexane interface. Systems M1 to M4 correspond to MPD-TMC systems with increasing MPD concentration from M1 to M3, followed by an increase in TMC concentration from M3 to M4 (Table S1). Water fingers (in yellow) are observed only in the M3 and M4 systems. Systems P1 to P4 represent PIP-TMC systems with progressively increasing monomer concentrations (Table S2), where water fingers are present in all P1-P4 systems. (**B** and **C**) Properties of the incipient polyamide films in MPD-TMC (B) and PIP-TMC (C) systems. From top to bottom: crosslinking degree, average pore size, free volume fraction and density. Enhanced water finger formation in the M2 system (**D**) upon the addition of acetone as a co-solvent in the *n*-hexane phase and (**E**) when micellar SDS solutions are added to the water phase. (**F**) Suppression of water fingers in the P3 system when a monolayer of SDS is introduced into the water phase.


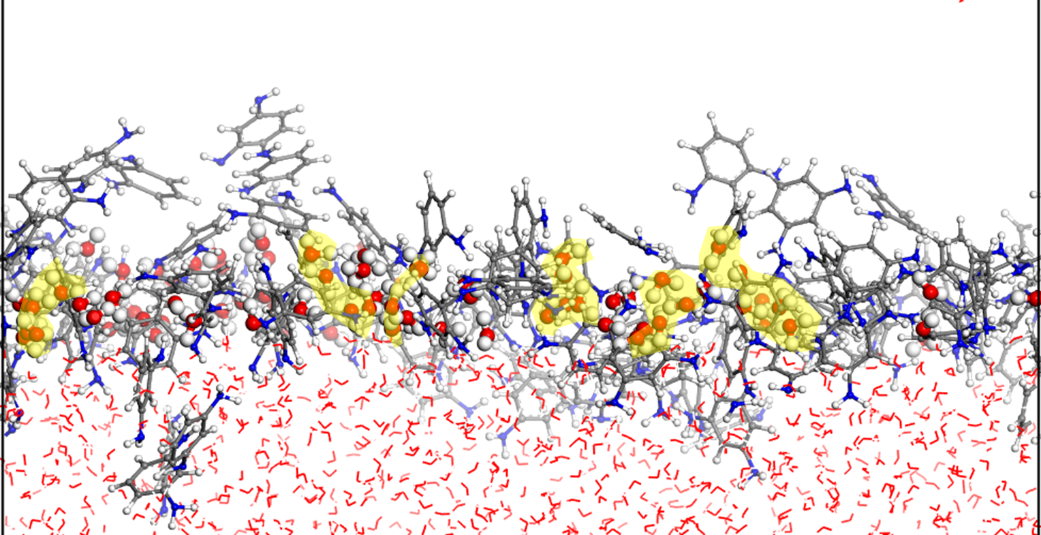


**Fig. S34. Snapshot of interfacial water and MPD molecules in M3 system (at 333 K) during 1 ns of equilibrium diffusion.** Water fingers are highlighted in yellow.

The formation of water fingers is closely associated with thermally excited capillary waves and interfacial fluctuations. To explore the influence of temperature, we conducted additional MD simulations on the MPD–TMC M3 system under elevated temperatures at 333K. As shown in Figure S34, thermal fluctuations became more prominent after 1 ns of dynamics, leading to more frequent and intense interfacial perturbations. These enhanced capillary waves facilitated the transient emergence of water fingers, supporting the notion that higher temperatures promote finger formation by increasing interfacial instability.

**Table S1. Molecular compositions of different MPD–TMC systems (M1–M4) and pure water/*n*-hexane system.**

| **System** | **water phase** | | | ***n*-hexane phase** | | |
| --- | --- | --- | --- | --- | --- | --- |
|  | Number of molecules | | Weight % | Number of molecules | | Weight % |
|  | water | MPD |  | *n*-hexane | TMC |  |
| **M1** | 4000 | 50 | 7.0 | 600 | 50 | 20.4 |
| **M2** | 4000 | 100 | 13.0 | 600 | 50 | 20.4 |
| **M3** | 4000 | 200 | 23.1 | 600 | 50 | 20.4 |
| **M4** | 4000 | 200 | 23.1 | 600 | 200 | 50.7 |
| **Pure** | 4000 | 0 | 0 | 600 | 0 | 0 |

**Table S2. Molecular compositions of different PIP–TMC systems (P1–P4).**

| **System** | **water phase** | | | ***n*-hexane phase** | | |
| --- | --- | --- | --- | --- | --- | --- |
|  | Number of molecules | | Weight % | Number of molecules | | Weight % |
|  | water | PIP |  | *n*-hexane | TMC |  |
| **P1** | 4000 | 50 | 5.6 | 600 | 50 | 20.4 |
| **P2** | 4000 | 100 | 10.7 | 600 | 50 | 20.4 |
| **P3** | 4000 | 200 | 19.3 | 600 | 50 | 20.4 |
| **P4** | 4000 | 200 | 19.3 | 600 | 200 | 50.7 |

**Table S3. Descriptions of thermodynamic ensembles applied in this work.**

| **Thermodynamic Ensemble** | **Description** |
| --- | --- |
| **NVE** | Constant total number of particles (*N*), the system's volume (*V*), and the total energy in the system (*E*) |
| **NVT** | Constant total number of particles (*N*), the system's volume (*V*), and the absolute temperature (T) |
| **NPT** | Constant total number of particles (*N*), constant pressure applied (*P*), and the absolute temperature (T) |

**Table S4. Eigenvalues of HOMO and LUMO for different reactive monomers.**

| **Monomer** | **HOMO** | **LUMO** |
| --- | --- | --- |
| **MPD** | -0.16 | -0.01 |
| **PIP** | -0.18 | 0.05 |
| **TMC** | -0.27 | -0.15 |

**Table S5. Diffusion coefficients of the targeted MPD molecule in different sub-processes during the 5 ns diffusion course presented in Fig. S17.**

| **Sub-process** | **I** | **II** | **III** |
| --- | --- | --- | --- |
| **Diffusion coefficient of MPD (m^2^ s^-1^)** | 5.6 × 10 ^-10^ | 1.6 × 10 ^-10^ | 1.4 × 10 ^-10^ |

**Table S6. Diffusion coefficients of the targeted PIP molecule in different sub-processes during the 5 ns diffusion course presented in Fig. S18.**

| **Sub-process** | **I** | **II** | **III** |
| --- | --- | --- | --- |
| **Diffusion coefficient of PIP (m^2^ s^-1^)** | 1.4 × 10 ^-9^ | 0.4 × 10 ^-9^ | 2.9 × 10 ^-9^ |

**Table S7. Diffusion coefficients (m^2^ s^-1^) of amine monomers in the two pure solvents presented in Fig. S21.**

| **Monomer** | **Diffusion coefficient in water (m^2^ s^-1^)** | **Diffusion coefficient in *n*-hexane (m^2^ s^-1^)** |
| --- | --- | --- |
| **MPD** | 9.8 × 10 ^-10^ | 3.7 × 10 ^-9^ |
| **PIP** | 2.2 × 10 ^-9^ | 3.1 × 10 ^-9^ |

**Table S8. Binding energies of MPD and PIP with both solvents.**

| **Binding energy (kcal mol^-1^)** | **water** | ***n*-hexane** |
| --- | --- | --- |
| **MPD** | -28.4 | -15.3 |
| **PIP** | -25.5 | -12.0 |

**Movie S1. Dynamic evolution of the interfacial region in M3 system by molecular dynamics (MD) simulations.** All water molecules and **one** MPD molecule that exhibits vigorous diffusion at the interface are shown in ball-and-stick models for clarity. The other MPD molecules are represented by line models. Other molecules are set invisible.

**Movie S2. Dynamic evolution of the interfacial region in P3 system by MD simulations.** All water molecules and **four** PIP molecules that exhibit vigorous diffusion at the interface and finally penetrate into the *n*-hexane phase are shown in ball-and-stick models for clarity. The other PIP molecules are represented by line models. Other molecules are set invisible.

**Movie S3. Dynamic evolution of the interfacial region in M4 system by MD simulations.** All water molecules and **four** MPD molecules that exhibit vigorous diffusion at the interface are shown in ball-and-stick models for clarity. The other MPD molecules are represented by line models. Other molecules are set invisible.

**Movie S4. Dynamic evolution of the interfacial region in P4 system by MD simulations.** All water molecules and **eleven** PIP molecules that exhibit vigorous diffusion at the interface and finally penetrate into the *n*-hexane phase are shown in ball-and-stick models for clarity. The other PIP molecules are represented by line models. Other molecules are set invisible.

**Movie S5. Dynamic evolution of the interfacial region in co-solvent M2 system by MD simulations.** All water molecules and **one** MPD molecule that exhibits vigorous diffusion at the interface and finally penetrate into the *n*-hexane phase are shown in ball-and-stick models for clarity. The other MPD molecules are represented by line models. Other molecules are set invisible. Compared with M2 system in Fig. S25, the co-solvent M2 system exhibit water fingers which are largely enhanced by the co-solvent acetone in the *n*-hexane phase.

**Movie S6. Dynamic evolution of the interfacial region in micellar M2 system by MD simulations.** All water molecules and **two** MPD molecules that exhibit vigorous diffusion at the interface and finally penetrate into the *n*-hexane phase are shown in ball-and-stick models for clarity. The other MPD molecules are represented by line models. Other molecules are set invisible. Compared with M2 system in Fig. S25, the micellar M2 system exhibit water fingers which are largely enhanced by the sodium dodecyl sulfate (SDS) monolayer and micelle.

**Movie S7. Dynamic evolution of the interfacial region in monolayered P3 system by MD simulations.** All water molecules and **eight** PIP molecules that exhibit vigorous diffusion at the interface are shown in ball-and-stick models for clarity. The other PIP molecules are represented by line models. Other molecules are set invisible. Compared with P3 system in Movie S2, the monolayered P3 system exhibit water fingers which are largely hindered by the SDS monolayer.

**Reference**

1. I. Benjamin, Chemical reactions and solvation at liquid interfaces: A microscopic perspective. *Chem. Rev.* **96**, 1449–1475 (1996). [doi: 10.1021/cr950230](https://doi.org/10.1021/cr950230)+
2. K. Piradashvili, E. M. Alexandrino, F. R. Wurm, K. Landfester, Reactions and polymerizations at the liquid-liquid interface. *Chem. Rev.* **116**, 2141–2169 (2016). [doi: 10.1021/acs.chemrev.5b00567](https://doi.org/10.1021/acs.chemrev.5b00567)
3. M. F. Ruiz-Lopez, J. S. Francisco, M. T. C. Martins-Costa, J. M. Anglada, Molecular reactions at aqueous interfaces. *Nat. Rev. Chem.* **4**, 459–475 (2020). [doi: 10.1038/s41570-020-0203-2](https://doi.org/10.1038/s41570-020-0203-2)
4. L. F. Scatena, M. G. Brown, G. L. Richmond, Water at hydrophobic surfaces: Weak hydrogen bonding and strong orientation effects. *Science* **292**, 908–912 (2001). [doi: 10.1126/science.1059514](https://doi.org/10.1126/science.1059514)
5. W. H. Steel, R. A. Walker, Measuring dipolar width across liquid–liquid interfaces with ‘molecular rulers.’ *Nature* **424**, 296–299 (2003). [doi: 10.1038/nature01791](https://doi.org/10.1038/nature01791)
6. P. Ball, How to keep dry in water. *Nature* **423**, 25–26 (2003). [doi: 10.1038/423025a](https://doi.org/10.1038/423025a)
7. I.-F. W. Kuo, C. J. Mundy, An ab Initio Molecular Dynamics Study of the Aqueous Liquid-Vapor Interface. *Science* **303**, 658–660 (2004). [doi: 10.1126/science.1092787](https://doi.org/10.1126/science.1092787)
8. A. Poynor, L. Hong, I. K. Robinson, S. Granick, Z. Zhang, P. A. Fenter, How water meets a hydrophobic surface. *Phys. Rev. Lett.* **97**, 1–4 (2006). [doi: 10.1103/PhysRevLett.97.266101](https://doi.org/10.1103/PhysRevLett.97.266101)
9. D. Chandler, Oil on troubled waters. *Nature* **445**, 831–832 (2007). [doi: 10.1038/445831a](https://doi.org/10.1038/445831a)
10. J. G. Davis, K. P. Gierszal, P. Wang, D. Ben-Amotz, Water structural transformation at molecular hydrophobic interfaces. *Nature* **491**, 582–585 (2012). [doi: 10.1038/nature11570](https://doi.org/10.1038/nature11570)
11. I. Benjamin, Mechanism and Dynamics of Ion Transfer Across a Liquid-Liquid Interface. *Science* **261**, 1558–1560 (1993). [doi: 10.1126/science.261.5128.1558](https://doi.org/10.1126/science.261.5128.1558)
12. N. Kikkawa, L. Wang, A. Morita, Microscopic Barrier Mechanism of Ion Transport through Liquid–Liquid Interface. *J. Am. Chem. Soc.* **137**, 8022–8025 (2015). [doi: 10.1021/jacs.5b04375](https://doi.org/10.1021/jacs.5b04375)
13. Y. Ghadar, P. Parmar, A. C. Samuels, A. E. Clark, Solutes at the liquid:liquid phase boundary—Solubility and solvent conformational response alter interfacial microsolvation. *J. Chem. Phys.* **142,** 104707 (2015). [doi: 10.1063/1.4914142](https://doi.org/10.1063/1.4914142)
14. A. Nilsson, L. G. M. Pettersson, The structural origin of anomalous properties of liquid water. *Nat. Commun.* **6**, 8998 (2015). [doi: 10.1038/ncomms9998](https://doi.org/10.1038/ncomms9998)
15. O. Björneholm, M. H. Hansen, A. Hodgson, L.-M. Liu, D. T. Limmer, A. Michaelides, P. Pedevilla, J. Rossmeisl, H. Shen, G. Tocci, E. Tyrode, M.-M. Walz, J. Werner, H. Bluhm, Water at Interfaces. *Chem. Rev.* **116**, 7698–7726 (2016). [doi: 10.1021/acs.chemrev.6b00045](https://doi.org/10.1021/acs.chemrev.6b00045)
16. P. W. Morgan, S. L. Kwolek, Interfacial polycondensation. II. Fundamentals of polymer formation at liquid interfaces. *J. Polym. Sci.* **40**, 299–327 (1959). [doi: 10.1002/pola.1996.816](https://doi.org/10.1002/pola.1996.816)
17. F. Zhang, J. Fan, S. Wang, Interfacial Polymerization: From Chemistry to Functional Materials. *Angew. Chemie Int. Ed.* **59**, 21840–21856 (2020). [doi: 10.1002/anie.201916473](https://doi.org/10.1002/anie.201916473)
18. V. Freger, G. Z. Ramon, Polyamide desalination membranes: Formation, structure, and properties. *Prog. Polym. Sci.* **122**, 101451 (2021). [doi: 10.1016/j.progpolymsci.2021.101451](https://doi.org/10.1016/j.progpolymsci.2021.101451)
19. Y. Zhang, H. Wang, J. Guo, X. Cheng, G. Han, C. H. Lau, H. Lin, S. Liu, J. Ma, L. Shao, Ice-confined synthesis of highly ionized 3D-quasilayered polyamide nanofiltration membranes. *Science* **382**, 202–206 (2023). [doi: 10.1126/science.adi9531](https://doi.org/10.1126/science.adi9531)
20. S. Karan, Z. Jiang, A. G. Livingston, Sub–10 nm polyamide nanofilms with ultrafast solvent transport for molecular separation. *Science* **348**, 1347–1351 (2015). [doi: 10.1126/science.aaa5058](https://doi.org/10.1126/science.aaa5058)
21. H. B. Park, J. Kamcev, L. M. Robeson, M. Elimelech, B. D. Freeman, Maximizing the right stuff: The trade-off between membrane permeability and selectivity. *Science* **356**, 1138–1148 (2017). [doi: 10.1126/science.aab0530](https://doi.org/10.1126/science.aab0530)
22. Z. Tan, S. Chen, X. Peng, L. Zhang, C. Gao, Polyamide membranes with nanoscale Turing structures for water purification. *Science* **360**, 518–521 (2018). [doi: 10.1126/science.aar6308](https://doi.org/10.1126/science.aar6308)
23. M. R. Chowdhury, J. Steffes, B. D. Huey, J. R. McCutcheon, 3D printed polyamide membranes for desalination. *Science* **361**, 682–686 (2018). [doi: 10.1126/science.aar2122](https://doi.org/10.1126/science.aar2122)
24. T. E. Culp, B. Khara, K. P. Brickey, M. Geitner, T. J. Zimudzi, J. D. Wilbur, S. D. Jons, A. Roy, M. Paul, B. Ganapathysubramanian, A. L. Zydney, M. Kumar, E. D. Gomez, Nanoscale control of internal inhomogeneity enhances water transport in desalination membranes. *Science* **371**, 72–75 (2021). [doi: 10.1126/science.abb8518](https://doi.org/10.1126/science.abb8518)
25. A. Nowbahar, V. Mansard, J. M. Mecca, M. Paul, T. Arrowood, T. M. Squires, Measuring Interfacial Polymerization Kinetics Using Microfluidic Interferometry. *J. Am. Chem. Soc.* **140**, 3173–3176 (2018). [doi: 10.1021/jacs.7b12121](https://doi.org/10.1021/jacs.7b12121)
26. P. S. Singh, P. Ray, Z. Xie, M. Hoang, Synchrotron SAXS to probe cross-linked network of polyamide ‘reverse osmosis’ and ‘nanofiltration’ membranes. *J. Memb. Sci.* **421**–**422**, 51–59 (2012). [doi: 10.1016/j.memsci.2012.06.029](https://doi.org/10.1016/j.memsci.2012.06.029)
27. W. H. Steel, R. A. Walker, Solvent Polarity at an Aqueous/Alkane Interface: The Effect of Solute Identity. *J. Am. Chem. Soc.* **125**, 1132–1133 (2003). [doi: 10.1021/ja028468k](https://doi.org/10.1021/ja028468k)
28. V. Freger, Kinetics of Film Formation by Interfacial Polycondensation. *Langmuir* **21**, 1884–1894 (2005). [doi: 10.1021/la048085v](https://doi.org/10.1021/la048085v)
29. I. L. Carpenter, W. J. Hehre, A molecular dynamics study of the hexane/water interface. *J. Phys. Chem.* **94**, 531–536 (1990). [doi: 10.1021/j100365a010](https://doi.org/10.1021/j100365a010)
30. D. M. Mitrinovic, Z. Zhang, S. M. Williams, Z. Huang, M. L. Schlossman, X-ray Reflectivity Study of the Water−Hexane Interface. *J. Phys. Chem. B* **103**, 1779–1782 (1999). [doi: 10.1021/jp984640o](https://doi.org/10.1021/jp984640o)
31. I. Nulens, R. Verbeke, T. Opsomer, J. Huang, Y. Wang, S. Caspers, A. Kubarev, A. H. McMillan, W. Dehaen, I. F. J. Vankelecom, Real-time monitoring of interfacial polymerization using fluorescent dyes. *J. Memb. Sci.* **686**, 121998 (2023). [doi: 10.1016/j.memsci.2023.121998](https://doi.org/10.1016/j.memsci.2023.121998)
32. Z. Jiang, S. Karan, A. G. Livingston, Water Transport through Ultrathin Polyamide Nanofilms Used for Reverse Osmosis. *Adv. Mater.* **30**, 1705973 (2018). [doi: 10.1002/adma.201705973](https://doi.org/10.1002/adma.201705973)
33. P. Sarkar, S. Modak, S. Karan, Ultraselective and Highly Permeable Polyamide Nanofilms for Ionic and Molecular Nanofiltration. *Adv. Funct. Mater.* **31**, 2007054 (2021). [doi: 10.1002/adfm.202007054](https://doi.org/10.1002/adfm.202007054)
34. Y. Wen, R. Dai, X. Li, X. Zhang, X. Cao, Z. Wu, S. Lin, C. Y. Tang, Z. Wang, Metal-organic framework enables ultraselective polyamide membrane for desalination and water reuse. *Sci. Adv.* **8**, 1–12 (2022). [doi: 10.1126/sciadv.abm4149](https://doi.org/10.1126/sciadv.abm4149)
35. Q. Shen, Q. Song, Z. Mai, K.-R. Lee, T. Yoshioka, K. Guan, R. R. Gonzales, H. Matsuyama, When self-assembly meets interfacial polymerization. *Sci. Adv.* **9** (2023). [doi: 10.1126/sciadv.adf6122](https://doi.org/10.1126/sciadv.adf6122)
36. Y. Liang, Y. Zhu, C. Liu, K.-R. Lee, W.-S. Hung, Z. Wang, Y. Li, M. Elimelech, J. Jin, S. Lin, Polyamide nanofiltration membrane with highly uniform sub-nanometre pores for sub-1 Å precision separation. *Nat. Commun.* **11**, 2015 (2020). [doi: 10.1038/s41467-020-15771-2](https://doi.org/10.1038/s41467-020-15771-2)
37. H. F. Ridgway, J. Orbell, S. Gray, Molecular simulations of polyamide membrane materials used in desalination and water reuse applications: Recent developments and future prospects. *J. Memb. Sci.* **524**, 436–448 (2017). [doi: 10.1016/j.memsci.2016.11.061](https://doi.org/10.1016/j.memsci.2016.11.061)
38. H. Zhang, M. S. Wu, K. Zhou, A. W.-K. Law, Molecular Insights into the Composition–Structure–Property Relationships of Polyamide Thin Films for Reverse Osmosis Desalination. *Environ. Sci. Technol.* **53**, 6374–6382 (2019). [doi: 10.1021/acs.est.9b02214](https://doi.org/10.1021/acs.est.9b02214)
39. J. He, J. R. McCutcheon, Y. Li, Effect of different manufacturing methods on polyamide reverse-osmosis membranes for desalination: Insights from molecular dynamics simulations. *Desalination* **547**, 116204 (2023). [doi: 10.1016/j.desal.2022.116204](https://doi.org/10.1016/j.desal.2022.116204)
